# Achieving over 200 Wh kg^−1^ sodium-ion pouch cell by quantitative engineering of hard carbon pores

**DOI:** 10.1093/nsr/nwaf566

**Published:** 2025-12-12

**Authors:** Zhihao Chen, Jialong Shen, Wenjie Deng, Yingshan Huang, Peizhao Shan, Yuhang Lou, Ling Li, Guanyin Gao, Yaxiong Yang, Shengnan He, Hongge Pan, Xianhong Rui, Yong Yang, Hai Yang, Yan Yu

**Affiliations:** Hefei National Research Center for Physical Sciences at the Microscale, iChEM (Collaborative Innovation Center of Chemistry for Energy Materials), Department of Materials Science and Engineering, CAS Key Laboratory of Materials for Energy Conversion, University of Science and Technology of China, Hefei 230026, China; Hefei National Research Center for Physical Sciences at the Microscale, iChEM (Collaborative Innovation Center of Chemistry for Energy Materials), Department of Materials Science and Engineering, CAS Key Laboratory of Materials for Energy Conversion, University of Science and Technology of China, Hefei 230026, China; Hefei National Research Center for Physical Sciences at the Microscale, iChEM (Collaborative Innovation Center of Chemistry for Energy Materials), Department of Materials Science and Engineering, CAS Key Laboratory of Materials for Energy Conversion, University of Science and Technology of China, Hefei 230026, China; Hefei National Research Center for Physical Sciences at the Microscale, iChEM (Collaborative Innovation Center of Chemistry for Energy Materials), Department of Materials Science and Engineering, CAS Key Laboratory of Materials for Energy Conversion, University of Science and Technology of China, Hefei 230026, China; State Key Laboratory for Physical Chemistry of Solid Surfaces, and Department of Chemistry, College of Chemistry and Chemical Engineering, Xiamen University, Xiamen 361005, China; Hefei National Research Center for Physical Sciences at the Microscale, iChEM (Collaborative Innovation Center of Chemistry for Energy Materials), Department of Materials Science and Engineering, CAS Key Laboratory of Materials for Energy Conversion, University of Science and Technology of China, Hefei 230026, China; Hefei National Research Center for Physical Sciences at the Microscale, iChEM (Collaborative Innovation Center of Chemistry for Energy Materials), Department of Materials Science and Engineering, CAS Key Laboratory of Materials for Energy Conversion, University of Science and Technology of China, Hefei 230026, China; Hefei National Research Center for Physical Sciences at the Microscale, iChEM (Collaborative Innovation Center of Chemistry for Energy Materials), Department of Materials Science and Engineering, CAS Key Laboratory of Materials for Energy Conversion, University of Science and Technology of China, Hefei 230026, China; Institute of Science and Technology for New Energy, Xi’an Technological University, Xi’an 710021, China; Institute of Science and Technology for New Energy, Xi’an Technological University, Xi’an 710021, China; Institute of Science and Technology for New Energy, Xi’an Technological University, Xi’an 710021, China; School of Materials and Energy, Guangdong University of Technology, Guangzhou 510006, China; State Key Laboratory for Physical Chemistry of Solid Surfaces, and Department of Chemistry, College of Chemistry and Chemical Engineering, Xiamen University, Xiamen 361005, China; Hefei National Research Center for Physical Sciences at the Microscale, iChEM (Collaborative Innovation Center of Chemistry for Energy Materials), Department of Materials Science and Engineering, CAS Key Laboratory of Materials for Energy Conversion, University of Science and Technology of China, Hefei 230026, China; Hefei National Research Center for Physical Sciences at the Microscale, iChEM (Collaborative Innovation Center of Chemistry for Energy Materials), Department of Materials Science and Engineering, CAS Key Laboratory of Materials for Energy Conversion, University of Science and Technology of China, Hefei 230026, China; National Synchrotron Radiation Laboratory, Hefei 230026, China

**Keywords:** sodium-ion batteries, hard carbon, biomass, closed pores

## Abstract

Energy-dense sodium-ion batteries (SIBs) offer lithium-free, cost-effective solutions for grid-scale energy storage. However, the structural complexity of hard carbon (HC) anodes hinders the establishment of a clear structure–performance relationship, leading to the insufficient performance of current HC when paired with advanced cathodes. In this study, we precisely adjusted the content and size of closed pores in HC using an economical, extensible rosin-assisted pore-promoting strategy, and quantified the effective pore volume for sodium storage through small-angle X-ray scattering experiments. We show that optimizing the closed pore size to increase the effective pore volume is key to enhancing the electrochemical performance of HC. By controlling the size (∼2 nm) of closed pores, we enhance the Na-cluster filled volume fraction of HC, resulting in an extended low-potential plateau (<0.1 V vs. Na^+^/Na) and higher sodium storage capacity. Additionally, we established a positive correlation between the plateau capacity of HC and the effective pore volume. Consequently, the 4.5 Ah pouch-type SIBs assembled with optimized HC here (areal capacity, 2.8 mAh cm^−2^) achieved a high energy density of 202 Wh kg^−1^, with over 80% capacity retention after 500 cycles at 0.5 C. This research provides a solution for realizing low-cost, advanced SIBs.

## Teaser

This work significantly improves the performance of sodium-ion batteries by designing the internal structure of the hard carbon material, providing a new solution for achieving low-cost, long-life and high-energy-density batteries.

## INTRODUCTION

The abundance and widely distributed sodium resources make sodium-ion batteries (SIBs) a feasible option for environmentally sustainable and cost-effective large-scale energy storage systems [1–4]. However, the commercialization of SIBs is still challenged by poor electrochemical performance and high costs, particularly for active materials. Significant efforts are being devoted to developing and optimizing cost-effective, high-performance electrode materials, including cathodes (layered transition metal oxides [5], Prussian blue analogs [6] and polyanionic compounds [7]) and anodes (hard carbon, HC), to advance the industrialization of SIBs. While significant progress has been achieved in cathode materials—particularly layered oxides demonstrating competitive specific capacities (>160 mAh g^−1^) and elevated average voltages (>3.2 V vs. Na⁺/Na)—with corresponding pouch-type full cells exceeding 165 Wh kg^−1^ [5,8], the development of high-energy-density SIBs remains constrained by a persistent challenge: the absence of high-performance HC anodes. Current anode limitations directly compromise energy density metrics, resulting in state-of-the-art SIBs still lagging behind commercial LiFePO_4_||graphite batteries (∼180 Wh kg^−1^) [9].

HC is a type of amorphous carbon composed of sp^2^ and sp^3^ hybridized carbon atoms, with a complex structure featuring pores, defects and curved graphene platelets [10,11]. The typical electrochemical curve of HC anodes can be divided into a slope region (>0.1 V vs. Na⁺/Na) and a plateau region (<0.1 V vs. Na⁺/Na), with the latter being a key factor determining the operating voltage and energy density of the full cell (Fig. S1) [12,13]. It has been noted that increasing the closed pore content of HC can enhance its plateau capacity, and many studies have focused on increasing the size and number of closed pores. For example, Li *et al.* [14] utilized chemical vapor deposition (CVD) of methane on porous carbon (PC) to increase sodium storage nanopore content, thereby promoting plateau capacity. The addition of pore-forming agents, such as CO_2_ [15], ethanol [16], MgO [17], KOH [18] and ZnO [19], has also been widely used as an effective strategy. However, the inherent structural complexity of HC often leads to insufficient control over the formation of closed pores, causing the electrochemical performance of HC to remain below expectations, particularly under high load conditions that fulfill the industrial requirements of SIBs [20–23]. Moreover, there is still a lack of fundamental understanding and rational design regarding the relationship between HC’s closed pore structure and sodium-storage behavior.

**Figure 1.**
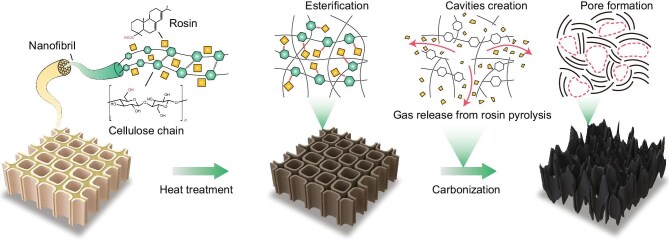
The mechanism of rosin for promoting microporous formation in biomass HC is attributed to the gases from rosin pyrolysis, which are key to creating spatial hindrance and cavities. The diagram only shows the cellulose chain, but the hydroxyl groups on hemicellulose and lignin can also esterify with rosin.

In this study, we utilized an esterification reaction to incorporate rosin acid into the polymer chains of biomass precursors (cellulose, hemicellulose and lignin). During subsequent aromatization and carbonization processes, the decomposition of rosin generates gases and creates spatial hindrance, allowing precise control over the closed pore structure of the HC (Fig. 1 and Fig. S2). By quantifying the effective pore volume of HC for sodium storage and the Na-cluster filled volume fraction, we demonstrated that the pore size is the key factor determining the Na^+^ pore-filling behavior. Specifically, smaller pore sizes (<2 nm) are favorable for the formation of Na clusters, and appropriately increasing defect concentration can help increase the Na-cluster filled volume fraction. Larger pore sizes (>2 nm) are detrimental to sodium storage, as larger Na clusters are thermodynamically unstable. In addition, we found that the plateau capacity of HC is directly proportional to the effective pore volume (the product of Na clusters filled volume fraction and closed pore volume). As a result, Pine HC with an average pore size of 1.91 nm exhibited the highest Na-cluster filled volume fraction (51.2%), corresponding to an effective pore volume of 0.033 cm^3^ g^−1^. The reversible sodium storage capacity reached 336 mAh g^−1^ (with 74.2% in the plateau region) and an initial Coulombic efficiency (ICE) of 92.2%, outperforming existing commercial HC. At high areal capacities (2.79/2.74 mAh cm^−2^), the 4.5 Ah pouch-type SIBs, with Pine HC as the anode and NaNi_1/3_Fe_1/3_Mn_1/3_O_2_ (NFM111) as the cathode, achieved a high energy density of over 202 Wh kg^−1^, with more than 80% capacity retention after 500 cycles.

## RESULTS AND DISCUSSION

### Pyrolysis of pine wood

**Figure 2.**
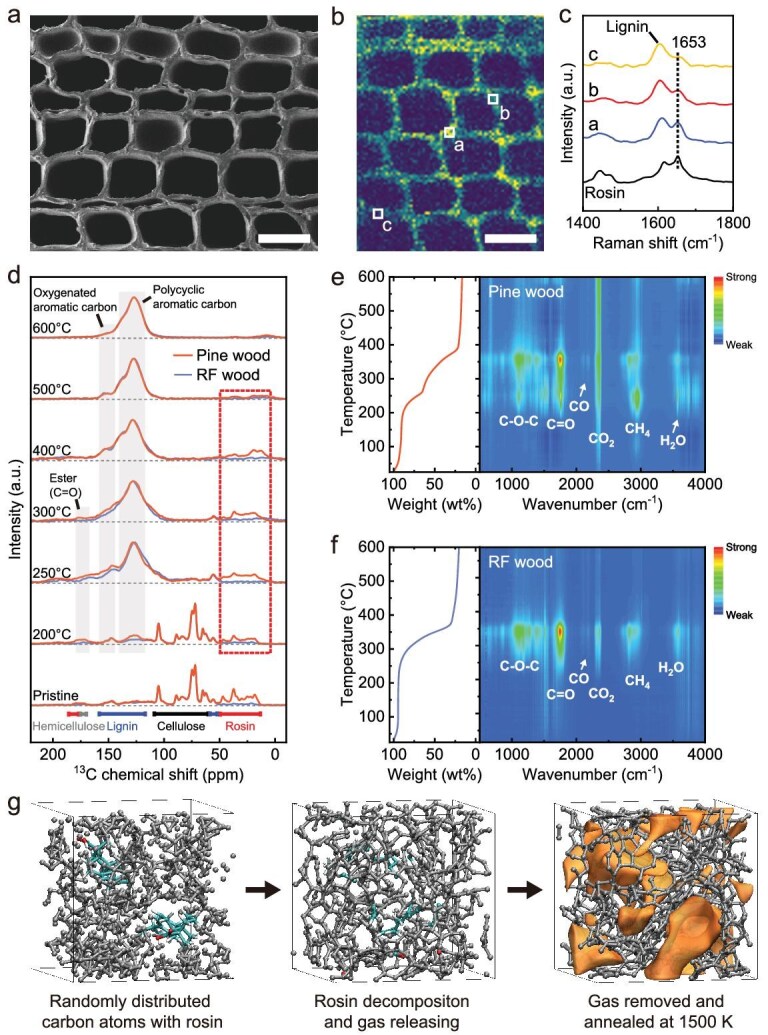
Pyrolysis mechanisms of pine wood. (a–c) SEM image, Raman mapping image and corresponding Raman spectra of pine wood. Raman color mapping is based on the C=C stretching vibration of rosin (black dashed line) at 1653 cm^−1^. Scale bars, 40 μm. (d) ^13^C CP/MAS solid-state NMR spectra of pine wood and RF wood at different pyrolysis temperatures. TGA-FTIR spectra of pine wood (e) and RF wood (f). The TGA curves are shown on the left and the FTIR spectra on the right are derived from the gases released during the pyrolysis of samples. (g) Snapshots of rosin pyrolysis and gas release from the carbon matrix, which induces the formation of microporous structures. The pores in the carbon matrix are shown in orange.

To elucidate the mechanism of the rosin-assisted pore-promoting strategy, we employed biomass containing rosin acid (e.g. waste pine wood) as a precursor for HC production as a demonstration. The distribution of rosin in pine wood was visualized using scanning electron microscopy (SEM) combined with Raman microscopy. The pine wood exhibited the characteristic porous structure typical of natural wood, with pore widths of approximately 10–40 μm (Fig. 2a). Raman mapping revealed that rosin was localized within these pore walls, permeating the entire matrix of the pine wood (Fig. 2b and c) (based on the C=C stretching vibration of rosin), with a content of about 3 wt% (Table S1). To explore the role of rosin in pyrolysis, the rosin-free (RF) wood was obtained by the Soxhlet extraction method (Fig. S3). The transverse relaxation distribution of the low-field nuclear magnetic resonance (LF-NMR) spectra of RF wood showed the absence of the rosin signal, indicating successful extraction (Fig. S4). Furthermore, the ^13^C solid-state NMR (ssNMR) spectra reveal the structural evolution of pine wood and RF wood during pyrolysis (Fig. 2d). After heat treatment, all samples were subjected to Soxhlet extraction to remove unreacted rosin, except for the pristine sample. The chemical shift signals of pristine-pine wood at 0–50 ppm are attributed to the C–H groups of rosin, whereas the signals corresponding to cellulose, hemicellulose and lignin are found in the 50–185 ppm range (Table S2) [24]. The 200°C pine wood sample retained degraded rosin, indicating that a portion of the rosin had been grafted onto the polymer chains and could not be extracted by solvent. Compared to 200°C RF wood, the 200°C pine wood exhibited stronger signals for ester carbon (173 ppm) and polycyclic aromatic carbon (128 ppm) [25]. This implies that esterification reactions occurred between rosin and the hydroxyl groups present in cellulose/hemicellulose/lignin, which compromised the thermal stability of the polymer chains, as confirmed by Fourier-transform infrared spectroscopy (FTIR) and X-ray diffraction (XRD) results (Figs S5 and S6). With an increase in carbonization temperature, cellulose and hemicellulose were observed to undergo pyrolysis and aromatization first (250°C–300°C). The ester signal of pine wood remained higher than that of RF wood. At 400°C, lignin and rosin were fully degraded and converted into polycyclic aromatic hydrocarbons and oxygenated aromatic hydrocarbons.

Further, we investigated the decomposition mechanism of pine wood and RF wood by thermogravimetric analysis coupled with FTIR (TGA-FTIR). The TGA curves of pine wood revealed a notable initial weight loss between 200°C and 280°C, primarily due to the esterification with rosin and the partial thermal degradation of cellulose, as evidenced by the gas signals (H_2_O, CO_2_ and CH_4_ etc.) detected in FTIR spectra (Fig. 2e and Table S3). The second weight loss occurred between 300°C and 400°C, signifying further pyrolysis of cellulose, hemicellulose and lignin, during which the carbon matrix underwent intramolecular cyclization and intermolecular aromatization [26]. During this stage, a substantial amount of the hydrogen and oxygen was removed, releasing more gases and organics compounds (ethers (C–O–C), aldehydes (C=O) and alkanes (C–C) etc.) [26]. Beyond 400°C, the weight change stabilized, although CO_2_ was continually released due to the cracking and reformation of carbonyl groups (C=O) after esterification, a process that facilitates the formation of the porous structure in the carbon matrix. In the case of RF wood, a significant mass loss solely occurred between 300°C and 400°C, attributable to the pyrolysis of polymer chains (Fig. 2f). The amount of CO_2_ released during subsequent carbonization was lower compared to pine wood. *Ab initio* molecular dynamics (AIMD) simulations were conducted to further elucidate the impact of rosin on the pore structure of the carbon matrix. Unlike pure carbon, the gases generated from rosin pyrolysis create spatial hindrance and form numerous cavities within the carbon network (Fig. 2g, Fig. S7 and Table S4). Even after the removal of these gases, the pores (highlighted in orange) remained stable during subsequent simulated annealing. These findings suggest that the esterification reaction with rosin altered the decomposition mechanisms of pine wood, thereby promoting the formation of the nanopore structure of HC.

### Characterization the structure of Pine HC

Pine wood and RF wood, treated at 200°C, were carbonized at a high temperature of 1300°C, then crushed and graded to produce Pine HC and RF HC, respectively (Fig. S8). In addition, to obtain HC with different closed pore structures, we prepared Pine HC variants with varying rosin content (Pine HC-*x*%, where *x* = 1, 6 or 10). XRD patterns revealed that all Pine HCs and RF HC are amorphous, as evidenced by broad (002) and (100) diffraction peaks, which is consistent with results of selected area electron diffraction (SAED) and wide-angle X-ray scattering (WAXS) (Figs S9–S11). Structure parameters are detailed in Table S5. Raman spectra showed that the I_D_/I_G_ value increased with the rise in rosin content (Fig. S12). The I_D_/I_G_ ratio increased from 1.80 in RF HC to 2.12 in Pine HC-10%, suggesting that the esterification with rosin enhances the defect concentration and reduces the graphitization degree of HC. This increase in structural disorder aids in the diffusion and storage of Na^+^ ions [27]. X-ray photoelectron spectroscopy (XPS) analysis further confirms that Pine HC-10% (6.4%) and Pine HC (5.8%) contain a higher oxygen content and more ester (−COOR) groups compared to RF HC (5.2%) (Fig. S13).

**Figure 3.**
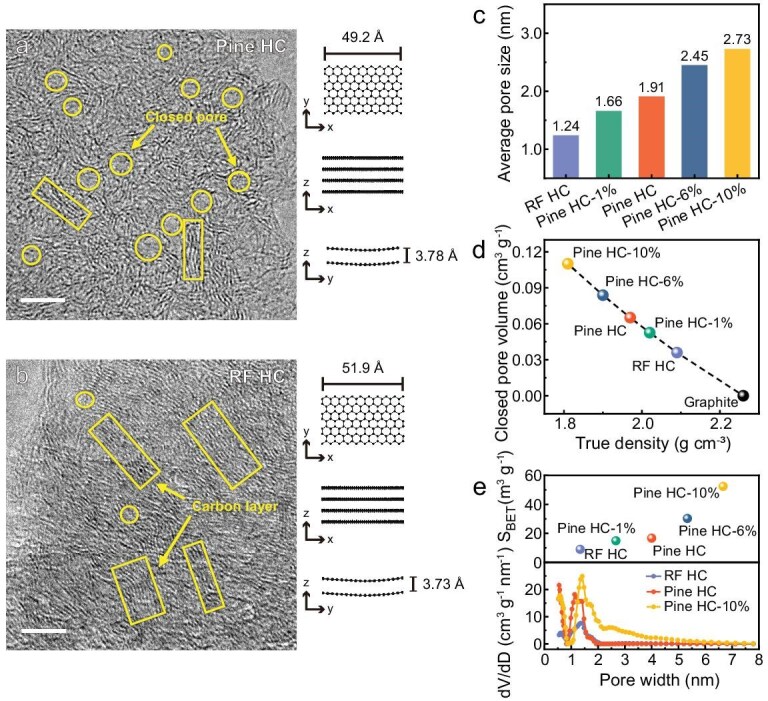
Pore structure characterization of Pine HCs and RF HC. HRTEM images of Pine HC (a) and RF HC (b). Scale bars, 5 nm. On the right side are the structures resulting from refinements against *L_a_, L_c_* and *d*_002_ of the HC. (c) The average closed pore size obtained from SAXS profiles. (d) True density (skeletal density) and the corresponding closed pore volume of Pine HCs, RF HC and graphite. (e) The BET surface area and the corresponding pore size distribution.

High-resolution transmission electron microscopy (HRTEM) reveals the microstructure of Pine HC and RF HC, both materials consisting of disorderly intertwined curved graphene platelets, which form nanocarbon layers and closed pores (Fig. 3a and b). Notably, Pine HC contains more and larger closed pores compared to RF HC, indicating that rosin promotes the formation of these nanopores. Additionally, we examined the nanopore structure (including open and closed pores) of all Pine HCs and RF HC using small-angle X-ray scattering (SAXS). Significant differences in scattering intensity at around 0.1 Å^−1^ were observed between the HCs (Fig. S14). Calculations based on the Teubner–Strey model reveal that Pine HC (1.91 nm) has a larger average pore diameter than RF HC (1.24 nm) (Fig. 3c and Table S6) [28], consistent with the TEM observations. As the rosin content reached 10% (Pine HC-10%), the average pore size further increased to 2.73 nm. As a complement to SAXS, we measured the true density of all Pine HCs and RF HC (Fig. 3d). The skeletal densities of Pine HC-10%, Pine HC-6%, Pine HC, Pine HC-1% and RF HC were 1.81, 1.90, 1.97, 2.02 and 2.09 g cm^−3^, respectively, with corresponding closed pore volumes of 0.110, 0.084, 0.065, 0.053 and 0.036 cm^3^ g^−1^. These findings suggest that the introduction of rosin results in larger closed pore sizes and volumes compared to RF HC. Furthermore, the Brunauer–Emmett–Teller (BET) surface area and open pore structure of HCs were characterized by nitrogen adsorption experiments. The rosin esterification influenced the surface structure of HC; Pine HC showed a BET surface area of 16.7 m^2^ g^−1^, in contrast to RF HC’s 9.0 m^2^ g^−1^ (Fig. 3e, Fig. S15 and Table S7). Pine HC-10%, with the highest rosin content, exhibits the largest BET surface area of 52.4 m^2^ g^−1^. The corresponding pore size distribution indicates that the higher specific surface area of Pine HC is mainly due to a greater number of pores in the 0.5–1.5 nm range compared to RF HC. It has been reported that these pores around 1 nm are difficult for the electrolyte to fully diffuse into, and wet, thus would not significantly affect the ICE of Pine HC [29]. In contrast, Pine HC-10% possesses more open pores in the 1.5−5 nm range than Pine HC, which could potentially exacerbate the irreversible decomposition of the electrolyte.

### Electrochemical performance of Pine HC

**Figure 4.**
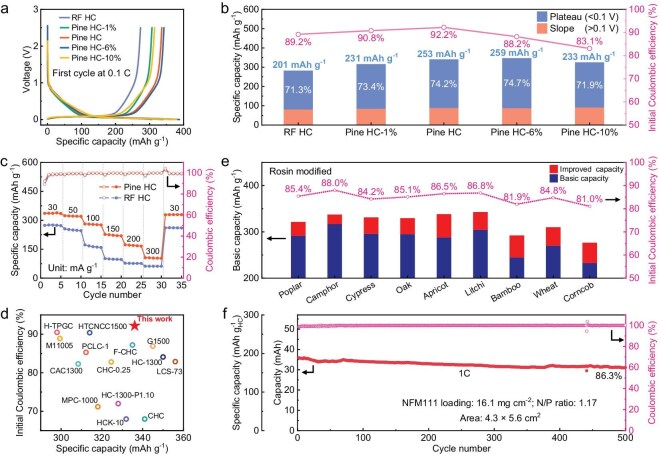
Electrochemical performance of Pine HCs and RF HC anodes. (a) Galvanostatic charge–discharge profiles of Pine HCs and RF HC at 30 mA g^−1^. (b) Comparison of the plateau and slope capacity ratio in the second discharge profiles. (c) Rate capability of Pine HC and RF HC under various current densities ranging from 30 to 300 mA g^−1^. (d) Comparison of the reversible capacity and ICE of Pine HC with the reported HC anodes. (e) Electrochemical performance of various biomass-derived rosin-modified HCs in terms of their reversible capacity and ICE. (f) Cycling performance of the NFM111||Pine HC pouch cell under a discharge current of 1 C.

The sodium storage performance of all Pine HCs and RF HC was evaluated in a half-cell configuration using an ester electrolyte (Fig. 4a, Figs S16 and S17). Pine HC-1% (with 1% rosin content) shows improved electrochemical performance compared to RF HC, with discharge capacity rising from 282 to 316 mAh g^−1^, and ICE increasing from 89.2% to 90.3%. Pine HC achieves a balance of high ICE (92.2%) and high capacity (341 mAh g^−1^). Pine HC-6% has the highest capacity at 347 mAh g^−1^, but its ICE drops to 88.2%. Due to irreversible Na^+^ loss caused by excessive specific surface area, the ICE of Pine HC-10% further decreases to 83.1%. A detailed analysis was conducted on the electrochemical performance differences among Pine HCs and RF HC, revealing that the increased plateau capacity is the main factor for the superior overall capacity of Pine HCs (Fig. 4b). As the rosin content increased (from 0% to 6%), the plateau capacity rose from 201 mAh g^−1^ in RF HC to 259 mAh g^−1^ in Pine HC-6%, an increase of 29%, with the corresponding plateau region percentage rising to 74.7%. Further increasing the rosin content to 10% resulted in a decrease in both reversible capacity and plateau capacity, which is due to excessive open pores and larger closed pores that are unfavorable for sodium storage. The rate performance of Pine HC (best overall electrochemical performance) and RF HC was further compared. Pine HC exhibited excellent rate performance and cycling stability, achieving reversible capacities of 322.6, 280.3, 224.9, 171.4 and 105.5 mAh g^−1^ at current densities of 50, 100, 150, 200 and 300 mA g^−1^, respectively (Fig. 4c and Fig. S18). At a current density of 100 mA g^−1^, Pine HC demonstrated an impressive cycling performance, achieving 400 cycles with a retention rate of 91.5%, indicating excellent cycling stability (Fig. S19). In addition, SEM images also showed that the structure of Pine HC remained intact after 400 cycles (Fig. S20).

The electrochemical kinetics of sodium storage in Pine HC and RF HC were investigated through galvanostatic intermittent titration technique (GITT) and cyclic voltammetry (CV) measurements. Analysis of GITT profiles indicated that, in the slope region (>0.1 V), Pine HC possessed higher Na^+^ ion diffusion coefficients (D_Na+_) compared to RF HC (Fig. S21). This suggests that the improved pore structure facilitates Na^+^ diffusion to the interlayer and edge sites of HC. In the plateau region (<0.1 V), the sodium storage mechanism transitions to closed pore filling (discuss later). This transition causes the D_Na+_ of Pine HC and RF HC to first decrease and then increase [30]. Moreover, CV curves at different scan rates demonstrate that Pine HC shows reduced voltage polarization and a higher capacitive contribution compared to RF HC (Fig. S22), reflecting faster Na^+^ diffusion kinetics. Owing to its pore-rich structure, Pine HC exhibits significant advantages in both specific capacity and ICE compared to previously reported high-performance HC anodes (Fig. 4d and Table S8) [18,31–45]. Additionally, this pore-promoting strategy has been applied to a variety of cellulose-based biomasses. By immersing raw materials (e.g. cypress wood, corncobs and wheat straw etc.) in an ethanol solution of rosin, followed by identical heat treatment and carbonization processes, we synthesized various biomass-derived HCs. Compared to the original samples, the rosin-modified samples showed a 10%–20% increase in capacity and a 3%–5% improvement in ICE (Fig. 4e, Fig. S23 and Table S9).

The Pine HC anode was subsequently paired with a homemade NFM111 cathode in a pouch cell configuration. SEM images show that the morphology of NFM111 consists of microspheres with diameters ranging from 5 to 8 μm (Fig. S24), and it delivers a reversible capacity of 140 mAh g^−1^ at 0.1 C, maintaining 78 mAh g^−1^ at 20 C (Fig. S25). Within a voltage range of 1.5–4 V, the NFM111||Pine HC pouch cell demonstrated a high ICE of 87.7% (Fig. S26), yielding an energy density of 244.4 Wh kg^−1^ (based on the total weight of active cathode and anode materials). Cyclic stability testing was conducted under conditions of 1 C discharge current and a voltage window of 1.5–3.8 V. The results showed that the capacity retention of NFM111||Pine HC pouch cell after 500 cycles was 86.3%, reflecting outstanding cycle stability (Fig. 4f and Fig. S27). Even at low temperatures of −10°C, the NFM111||Pine HC pouch cell retains an impressive capacity retention rate of 77.5% after 100 cycles (Fig. S28). Furthermore, the compatibility of the Pine HC anode with various cathodes, including Na_3_V_2_(PO_4_)_3_ (NVP), Na_4_Fe_3_(PO_4_)_2_P_2_O_7_ (NFPP), and Na_2_MnFe(CN)_6_ (PBA), was evaluated in pouch cells. The NVP||Pine HC, NFPP||Pine HC and PBA||Pine HC pouch cells exhibited impressive electrochemical performance, manifesting ICEs of 80.5%, 80.0% and 79.1%, and energy densities of 201.1, 165.1 and 258.8 Wh kg^−1^, respectively (Figs S29–S31). After 200 cycles, their capacity retentions were 82.5%, 81.5% and 80.0%, respectively (Figs S32–S34). These results indicate that Pine HC can be effectively paired with various cathode materials, showcasing its excellent potential for practical applications.

### Structural evolution during the first sodiation of Pine HC

**Figure 5.**
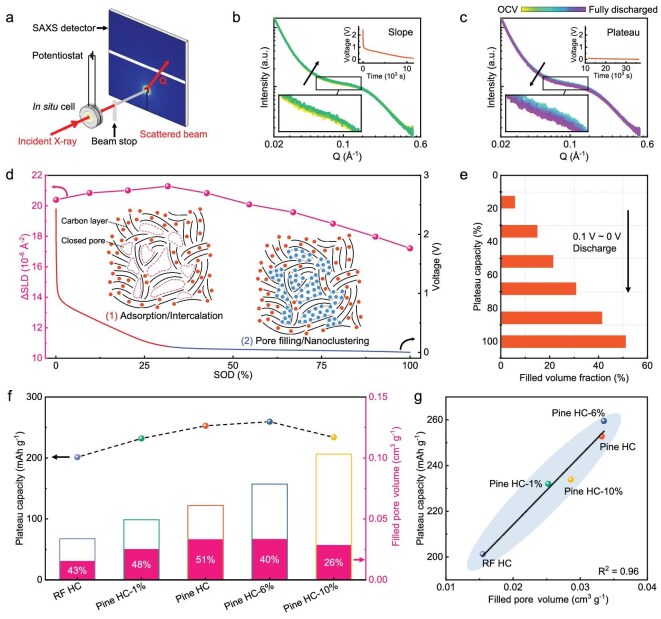
*In situ* SAXS studies for sodium storage mechanism in Pine HC anodes. (a) Schematic illustration of the experimental set-up for *in situ* SAXS. *In situ* SAXS profiles of the Pine HC electrode during the slope capacity region (b) and the plateau capacity region (c) for the first sodiation. Curves with various colors represent different discharge states. (d) Magnitude of the average ΔSLD between the carbon matrix and the closed pores upon different SODs and the corresponding discharge curves. Insert: schematic diagram of sodium storage stages in the slope and plateau capacity regions of Pine HC, where red spheres indicate adsorbed or intercalated Na^+^, and blue spheres indicate pore-filled Na^+^. (e) Calculated Na-cluster filled volume fraction in the plateau region of Pine HC. (f) Effective pore volumes of HCs determined from *in situ* SAXS experiments. (g) Linear fit between the effective pore volume and the plateau capacity of HCs.

To explore the structural evolution of Pine HC during the first discharge process, *in situ* SAXS experiments were performed (Fig. 5a and Fig. S35). The scattering intensity is directly related to the square of the difference in scattering length density (ΔSLD) between the carbon matrix and the pores. Therefore, SAXS can determine whether Na^+^ ions are intercalating into the carbon matrix or filling the closed pores. As sodiation progresses, the scattering intensity of the closed pores in Pine HC initially exhibits a slight increase (slope region) followed by a significant decrease (plateau region) (Fig. 5b and c). For quantification and comparison, ΔSLD between the carbon matrix and the pores at different states of discharge (SODs) was obtained by fitting SAXS data. The sodium storage mechanism of Pine HC can be divided into two stages: before reaching 30% SOD (the slope region), the ΔSLD increased from 20.4 × 10^−6^ to 21.3 × 10^−6^ Å^−2^, which indicates that Na^+^ adsorption/intercalation enhances the SLD of the carbon matrix, as the SLD of closed pores is zero (Fig. 5d) [14]. From 30% to 100% SOD (the plateau region), ΔSLD drops from 21.3 × 10^−6^ to 17.2 × 10^−6^ Å^−2^, suggesting that Na^+^ ions enter and fill the closed pores. These two stages were also captured by *in situ* XRD and *in situ* Raman experiments (Figs S36 and S37). In the slope region of sodiation, Pine HC exhibited a weakening of the (002) diffraction peak and a red shift of the G-band, disclosing that Na^+^ ion adsorb/intercalate into the carbon layers and electrons occupy the π* anti-bonding band of the graphene platelets [46]. These characteristic phenomena of sodium ion intercalation into carbon layers were not observed in some reports, which may be attributed to structural differences between Pine HC and the materials studied in those works [47,48]. For the plateau region, these phenomena are less pronounced, suggesting that Na^+^ intercalation is nearly saturated at this stage. During desodiation, the scattering intensity of Pine HC rises and nearly returns to its initial level, indicating high reversibility of sodium storage within the closed pores (Fig. S38).

*Ex situ*  ^23^Na ssNMR further investigated the changes in the chemical state of Na. A sharp peak near −10 ppm was observed, corresponding to the diamagnetic Na in the remaining electrolyte and the solid electrolyte interphase (SEI) layer (Fig. S39) [49]. As more Na fills the closed pores (0.01–0 V), signals indicating quasi-metallic Na clusters appear, shifting to higher chemical shifts (from 868 to 925 ppm). This is due to the increasing contribution of the Knight shift, which indicates the growing metallic character of Na [50]. Additionally, the presence of Na clusters was visualized by reacting sodiated Pine HC with a 1% phenolphthalein ethanol solution. When the electrode containing Na clusters is placed in the solution, bubbles (of H_2_) form and sodium ethoxide is produced, turning the solution red (Fig. S40). As the discharge depth increases, the redness of the solution deepens, suggesting an increase in the content of quasi-metallic Na clusters in the electrodes (Fig. S41).

### Effective pore volume and plateau region capacity

To investigate the relationship between sodium storage capacity and closed pore structure, we conducted *in situ* SAXS experiments on all Pine HC samples and RF HC, and calculated their Na-cluster filled volume fraction after sodiation (Fig. 5e, Fig. S42). As the closed pore size increases [from 1.24 nm (RF HC) to 2.73 nm (Pine HC-10%)], the Na-cluster filled volume fraction initially rises and then falls. An average pore size of 2 nm seems to be a critical point (Fig. S43); for small closed pores (<2 nm), the filled volume fraction increased with pore size (from 43.1% for RF HC to 51.2% for Pine HC). However, as pore size increased further (>2 nm), the filled volume fraction dropped significantly (26% for Pine HC-10%). We attribute this to two thermodynamic factors: HC defect concentration (I_D_/I_G_); and Na cluster stability [51]. In HCs with small closed pores (RF HC, Pine HC-1% and Pine HC), stable Na clusters form, and defect concentration induced by rosin enhances the filled volume fraction. Conversely, larger Na clusters are thermodynamically unstable, making formation difficult in HCs with large closed pores (Pine HC-6% and Pine HC-10%). To prove this, we calculated the formation energy of Na clusters (Na_n_) within closed pores and found that the average formation energy decreases significantly in magnitude as the cluster size increases (Figs S44 and S45). For smaller clusters, the formation energy is notably negative (less than −0.1 eV), indicating strong stability. However, for larger clusters like Na_59_ and Na_140_, the formation energy becomes only marginally negative (approximately −0.05 eV). This very low energy gain implies minimal thermodynamic driving force for the formation of such large clusters at room temperature (*k*_B_T ∼ 0.026 eV), rendering them thermodynamically unstable within the confined pores. Therefore, samples with larger closed pores (Pine HC-6% and Pine HC-10%) showed poor cycling stability (Fig. S46).

Furthermore, we define these fillable closed pore volumes as effective pore volume (effective pore volume = filled volume fraction × closed pore volume) (Fig. 5f). It is evident that the effective pore volume, rather than the entire closed pore volume of HC, provides the sodium storage capacity. By comparing the effective pore volume and the plateau capacity of HCs, we found a strong correlation between the two (R^2^ > 0.96) (Fig. 5g). Effective pore volume determines Na^+^ plateau storage, indicating that optimizing closed pore size to increase filled volume fraction is crucial for improving HC electrochemical performance.

### Pilot production of Pine HC and high-energy pouch cells

**Figure 6.**
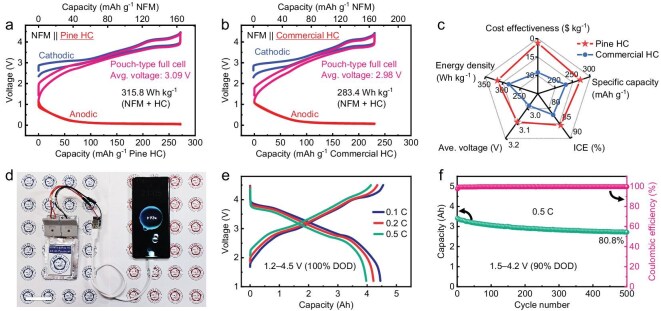
The advantages of Pine HC over commercial HC and the high energy density achieved in Ah-level pouch cells. Electrochemical profiles of NFM111||Pine HC (a) and NFM111||commercial HC (b) measured in a three-electrode pouch cell within 1.2–4.5 V with a negative/positive (N/P) capacity ratio of 1.12 (reference electrode: Na metal). (c) Radar chart comparing the electrochemical performance in the pouch cell and the cost-effectiveness of Pine HC and commercial HC. 4.5 Ah-laminated NFM111||Pine HC pouch cell with an energy density of 202 Wh kg^−1^: photograph showing that it successfully powered a smartphone to 93% capacity (d), the charge–discharge profiles within 1.2–4.5 V (e), and the cycling performance at 0.5 C within 1.5–4.2 V (f). Scale bar, 5 cm.

As a proof of concept, 3 kg of Pine HC was produced using pine sawdust (Fig. S47). The estimated production cost is $3.53 kg^−1^ (Table S10), which is only 13% of the current commercial HC price (Kuraray Type 2, $27.5 kg^−1^) and significantly lower than the low-cost synthesis HC benchmark ($5 kg^−1^) [52]. Additionally, Pine HC and commercial HC were compared in a three-electrode pouch cell within the voltage window of 1.2–4.5 V (Fig. 6a and b, Figs S48 and S49). The red and blue profiles illustrate the potential responses of the anode and cathode against the reference electrode (Na metal) under full cell conditions, respectively. Upon reaching the cut-off voltage, the cathode paired with Pine HC exhibited a lower potential of 4.53 V compared to that paired with commercial HC (4.57 V), thus reducing the risk of irreversible phase transitions, gas evolution and electrolyte decomposition in the pouch cell [8,53]. More importantly, the NFM111||Pine HC cell achieved a specific energy density of 315.8 Wh kg^−1^ (average voltage: 3.09 V), surpassing the 283.4 Wh kg^−1^ (2.98 V) obtained by NFM111||commercial HC cell (based on the total weight of active cathode and anode materials). The radar chart comparison highlights the advantages of Pine HC over commercial HC (Fig. 6c and Table S11). Furthermore, we fabricated a 4.5 Ah-laminated NFM111||Pine HC pouch cell with an energy density of 202 Wh kg^−1^ (based on the weight of the entire cell) (Fig. 6d and e, Fig. S50). Detailed information about this cell is provided in Table S12. After 500 cycles at 90% depth of discharge (DOD), the capacity retention remains above 80% (Fig. 6f and Fig. S51). These results clearly demonstrate that Pine HC holds significant potential for industrial applications in SIBs.

## CONCLUSIONS

To summarize, we developed an economical, extensible rosin-assisted pore-promoting strategy to precisely engineer the content and size (1.2–2.7 nm) of nanopores in HC. Based on this, we deconstructed the relationship between sodium storage capacity and closed pore structure, demonstrating that pore size is the critical parameter governing Na filling; HC with smaller closed pore sizes (<2 nm) can increase the Na-cluster filled volume fraction by enhancing defect concentration, while HC with larger closed pores (>2 nm) hinders Na-cluster formation, resulting in a lower Na-cluster filled volume fraction. By optimizing defect concentration and closed pore size, the Na-cluster filled volume fraction of the obtained Pine HC exceeds 50%, with a reversible sodium storage capacity of 336 mAh g^−^¹ and an ICE of 92.2%. Furthermore, the fabricated 4.5 Ah-laminated NFM111||Pine HC pouch cell achieved a high energy density (exceeding 202 Wh kg^−1^) and exhibited excellent cycling stability, with a capacity retention of 80.8% after 500 cycles. This work represents a promising development path for cost-effective, high-performance anodes for large-scale energy storage systems.

## Supplementary Material

nwaf566_Supplemental_File
