# Supplementary material for: Achieving over 200 Wh kg−1 sodium-ion pouch cell by quantitative engineering of hard carbon pores
Source: Natl Sci Rev. 2025 Dec 12;13(3):nwaf566. doi: 10.1093/nsr/nwaf566 (PMC12875110; doi:10.1093/nsr/nwaf566)
Supplement: nwaf566_Supplemental_File [file nwaf566_supplemental_file.pdf]

Achieving over 200 Wh kg^{-1} Sodium-Ion Pouch Cell by Quantitative Engineering of Hard Carbon Pores

Zhihao Chen,[1,#] Jialong Shen,[1,#] Wenjie Deng,[1,#] Yingshan Huang,[1] Peizhao Shan,[4] Yuhang Lou,[1] Ling Li,[1] Guanyin Gao,[1] Yaxiong Yang,[5] Shengnan He,[5] Hongge Pan,[5] Xianhong Rui,[3] Yong Yang,[4] Hai Yang,[1,*] and Yan Yu[1,2,*]

[1]Hefei National Research Center for Physical Sciences at the Microscale, iChEM (Collaborative Innovation Center of Chemistry for Energy Materials), Department of Materials Science and Engineering, CAS Key Laboratory of Materials for Energy Conversion, University of Science and Technology of China, Hefei 230026, China;

[2]National Synchrotron Radiation Laboratory, Hefei 230026, China;

[3]School of Materials and Energy, Guangdong University of Technology, Guangzhou 510006, China;

[4]State Key Laboratory for Physical Chemistry of Solid Surfaces, and Department of Chemistry, College of Chemistry and Chemical Engineering, Xiamen University, Xiamen 361005, China;

[5]Institute of Science and Technology for New Energy, Xi'an Technological University, Xi'an 710021, China

***Corresponding authors**. E-mails: yanghai@ustc.edu.cn; yanyumse@ustc.edu.cn

[#]Equally contributed to this work.

Methods

Synthesis of HC samples

To prepare Pine HC, 50 g of pine waste wood (with 3 wt% rosin content) (Huadong Timber Market, Jinhua, Zhejiang) was first placed in a muffle furnace and heated at 200 °C for 6 hours. Subsequently, it was carbonized at 1300 °C for 2 hours in a box furnace under a nitrogen atmosphere. The resulting material was then ground using a jet mill and sieved through a 1000-mesh sieve to obtain Pine HC. The rosin was extracted from the pine wood using a Soxhlet extractor to produce rosin-free wood (RF wood), which was then subjected to the same process to obtain RF HC. RF wood was soaked in a rosin ethanol solution containing 1 wt%, 6wt% and 10 wt% rosin relative to the RF wood. After solvent removal, the wood underwent heat treatment, carbonization, and pulverization to produce Pine HC-1%, Pine HC-6% and Pine HC-10%. Other biomass precursors were also soaked in a rosin ethanol solution containing 3 wt% rosin of the precursor and then processed similarly to yield rosin-modified HCs. Commercial HC was obtained from Kuraray Co. Ltd., Japan.

Electrochemical measurements

The slurry containing active material, carbon black, and sodium carboxymethylcellulose (mass ratio 92:3:5) was coated on Cu foil to prepare HC electrodes, with deionized water as the solvent and maintaining an active material mass loading of 5 mg cm^{-2}. The coin-type cells (CR2032) were assembled in an argon-filled glove box using sodium foil, glass fiber, and 1M $NaPF_6$ solution in a ethylene carbonate (EC) and dimethyl carbonate (DMC) (1:1 by volume) as the counter electrode, separator, and electrolyte, respectively. Galvanostatic charge-discharge and GITT tests were performed using a Neware battery testing system (CT-4008T-5 V10 mA-164, Shenzhen, China) within a voltage range of 0−2.5V.

The NFM111 cathode for pouch cells was purchased from Guangdong Canrd Co., Ltd., while the NVP, NFPP, and PBA cathodes were obtained from Hefei Kejing Co., Ltd. Both cathode and anode utilized Al foil as current collectors, with the cathode

measuring 4.3×5.6 cm^2 and the anode measuring 4.5×5.8 cm^2. The electrolyte consisted of a 1M $NaPF_6$ solution in a mixture of EC, propylene carbonate (PC), DMC, and ethyl methyl carbonate (EMC) (1:1:4:4 by volume) with 3% fluoroethylene carbonate (FEC) additive. A 12 μm polyethylene (PE) separator was employed.

Three-electrode pouch cell was fabricated following the method described by Li et al.[1] and was evaluated using an electrochemical workstation (PARSTAT 4000A).

Materials characterization

The morphologies and structure of the samples were examined using SEM (CIQTEK SEM3100) and TEM (JEOL, 2100F). XRD analyses were conducted with a Rigaku, TTR-III diffractometer utilizing Cu Kα radiation, with in-situ XRD patterns recorded every 7 minutes. Raman spectra and mappings were obtained via a WITec alpha300 R Raman imaging microscope equipped with a 532 nm excitation laser, with in-situ Raman spectra collected every 10 minutes. XPS measurements were performed on a Thermo ESCALAB 250Xi spectrometer. TGA-FTIR (PerkinElmer TL-9000) was carried out in a N_2 atmosphere at a heating rate of 10 °C min^{-1}. The true density of the samples was determined using a JW-M100A analyzer with He as the analysis gas. ^{13}C and ^{23}Na ssNMR spectra were acquired at room temperature on a Bruker AVANCE III 400 WB spectrometer. For ex situ experiments, electrodes were collected from Cu foil, transferred into 1.3 mm rotors without rinsing, and sealed with Vespel caps to minimize air or moisture exposure. SAXS experiments were conducted on a SAXSpoint 2.0 equipped with a 50 W micro-focus X-ray source (1 mm × 1 mm). The Teubner-Strey model was applied using SasView 5.0.6 software to analyze and obtain nanopore structural information[2]. During in-situ SAXS experiments, data were acquired every 30 minutes. Background data were obtained using a blank in-situ cell, and the measured signal intensity was calibrated using glassy carbon as a reference standard. Based on the measured SLD (scattering length density) of closed pores, the porosity of Pine HC, and the density of metallic Na, we estimated the filled volume fraction of Na clusters in HC[3].

Calculation of structure parameters (L_a, L_c, and n)

The stacking distance and longitudinal size of the carbon layers are calculated using the following formula (Scherrer equation):

$$L_{\mathrm{a}} = \frac{1.84\lambda}{\beta\cos\theta} \qquad (1)$$

$$L_c = \frac{0.90\lambda}{\beta\cos\theta} \qquad (2)$$

where λ represents the X-ray wavelength. β and θ represent the half-width at half-maximum (FWHM) and angle of the crystal plane signal peak ((002) or (100)), respectively. L_a takes the (100) crystal plane, and L_c takes the (002) crystal plane.

The number of stacking layers is calculated using the following formula:

$$n = \frac{L_c}{d_{002}} + 1 \qquad (3)$$

Calculation of closed pores parameter based on the Teubner-Strey model through SAXS results.

Referring to the method described by Saurel et al.[2], the closed pores size of hard carbon was calculated using the Teubner-Strey model:

$$I_{mp} = I_0 \frac{1}{1+C_1Q^2+C_2Q^4} \qquad (4)$$

where I_{mp} is the scattering intensity, and I_0、 C_1 and C_2 are defined by the following formular respectively:

$$I_0 = \frac{8\pi}{\rho_{struc}} \Phi(\Delta SLD)^2 \frac{\xi^3}{(1+(\frac{2\pi\xi}{d})^2)^2} \qquad (5)$$

$$C_1 = \frac{-2\xi^2(\frac{2\pi\xi}{d})^2+2\xi^2}{(1+(\frac{2\pi\xi}{d})^2)^2} \qquad (6)$$

$$C_2 = \frac{\xi^4}{(1+(\frac{2\pi\xi}{d})^2)^2} \qquad (7)$$

where d is the average distance between adjacent pores, and ξ is the relevant length that restricts long-range ordered expansion. The average closed pores size (D) can be calculated by the following formula:

$$D = 2\sqrt{5C_1} \qquad (8)$$

Calculation of Na clusters filled volume fraction and effective pore volume

We performed quantitative analysis using scattering length density (SLD) for hard carbons at different discharge states in plateau region. First, upon discharging to 0.1 V (end of the slope region), we assumed that the closed pores were not yet filled, resulting in a zero SLD contribution from them. Thus, the measured SLD change (ΔSLD) was entirely attributed to the hard carbon skeleton itself, i.e., $\Delta SLD = SLD_{sodiated\ hard\ carbon}$. Subsequently, at the end of the plateau region, we considered the capacity in this interval to be primarily due to the filling of closed pores by sodium clusters. Based on this, the SLD change in the plateau region was quantified as $\Delta SLD = SLD_{sodiated\ hard\ carbon} - SLD'_{closed\ pore}$, where $SLD'_{closed\ pore}$ represents the effective SLD of the filled closed pores. The theoretical SLD value (SLD_{Na}) and mass density for metallic sodium were obtained using the SLD calculator in the SasView software. Theoretically, if the closed pores are completely filled with sodium (i.e., 100% filling volume fraction), then $SLD'_{closed\ pore}$ should equal SLD_{Na}. In practice, the corresponding $SLD'_{closed\ pores}$ and mass density at the fully discharged state were derived from the in-situ SAXS signal intensity. By comparing these values with the theoretical values of metallic sodium, the actual filling volume fraction and effective pore volume were calculated.

Calculation of true density

The true density calculation was performed using the method described by Li et al.[4] Helium (He) gas was used as the detector for true density analysis, providing accurate values for the skeletal density, as He can penetrate almost all open pores except for closed ones. Graphite material is considered the densest carbon material without closed pores, with a theoretical true density value of up to 2.26 g·cm^{-3}. Therefore, using 2.26 g·cm^{-3} as the reference density, the closed pore volume of hard carbon materials can be calculated using the following formula:

$$V_{Close\ Pore} = \frac{1}{\rho_{true}} - \frac{1}{2.26} \tag{9}$$

Calculation methods

We used Packmol software to randomly put the rosins and C atoms into a 20×20×20 Å^3 box[5]. The Ab-initio Molecular Dynamics (AIMD) calculations were carried out by using CP2K software[6]. Goedecker-Teter-Hutter (GTH) pseudopotentials were used to

describe the core electrons and the exchange-correlation effects were represented by Perdew-Burke-Ernzerhof (PBE) functional[7-8]. The basis sets of H atom, C atom and O atom were DZVP-MOLOPT-SR-GTH-q1, DZVP-MOLOPT-SR-GTH-q4 and DZVP-MOLOPT-SR-GTH-q6, respectively. The energy cut-off was 300 Ry and energy convergence criterion was set to 1×10^{-5} Hartree. We used constant-volume (NVT) ensemble with a CSVR thermostat and opened OT method during the AIMD simulation[9]. The structure was first annealed at 2000 K with 0.3 fs time step and then annealed at 1500 K with 1.0 fs time step after removing rosins. The free volume and surface area of the final models were calculated and visualized using Multiwfn and VMD.

The formation energy for Na clusters of different sizes was simulated by placing Na clusters of varying dimensions between carbon layers spaced 20 nm apart. The formation energy was calculated using the following formula:

$$E_{formation} = \frac{(E_{C@Nax} - E_C - E_{Nax})}{x} \quad (10)$$

where x = 1, 4, 14, 59, 140. Here, $E_{C@Nax}$, E_C, and E_{Nax} represent the energy of the carbon layer with formation sodium clusters, the energy of the carbon layer, and the energy of the sodium clusters, respectively.

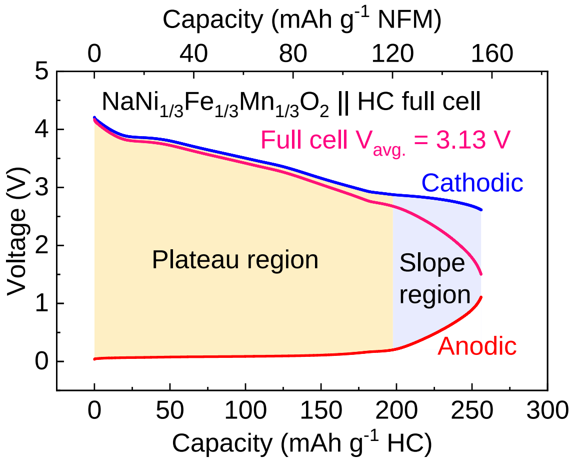

Figure S1: Electrochemical profiles of the $NaNi_{1/3}Fe_{1/3}Mn_{1/3}O_2$ (NFM111)||HC full cell measured in three-electrode cell within 1.2−4.2 V (reference electrode: Na metal). For a typical full cell, the voltage difference between the cathode and anode determines its discharge voltage. It is desirable for most of the discharge process to occur within the plateau region of HC, with only a small portion in the sloping capacity region, in order to enhance the energy density of the battery.

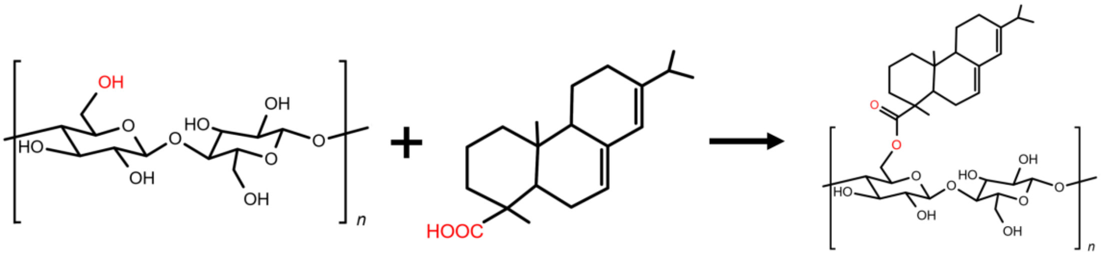

Figure S2: Schematic diagram of the esterification reaction between cellulose and rosin acid. The esterification reaction of the primary hydroxyl group in C6 position of cellulose with rosin acid is taken as an example because the esterification reactivity of C6−OH is the highest. Additionally, the hydroxyl groups on hemicellulose and lignin can also undergo esterification reactions with rosin acid.

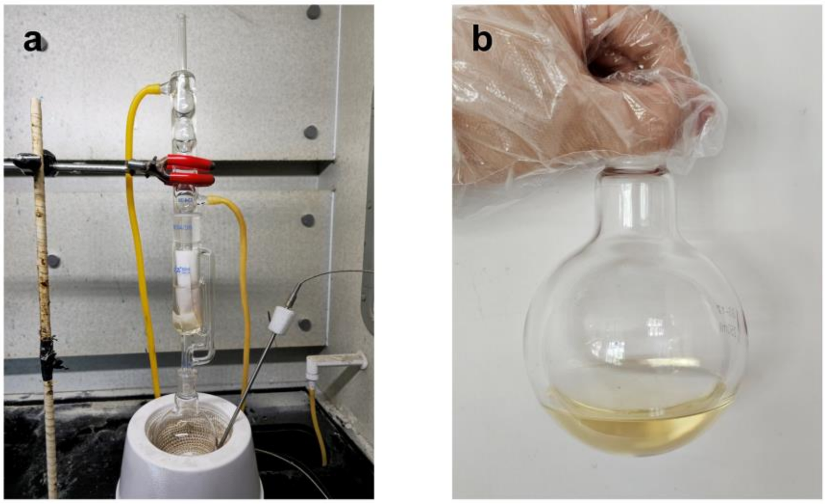

Figure S3: Optical photographs of Soxhlet extractor (**a**) and rosin extract (**b**). Soxhlet extraction uses the principles of solvent reflux and siphoning to continuously extract solid matter using a pure solvent. The solvent we used was a benzene-ethanol mixture (2:1 by volume ratio).

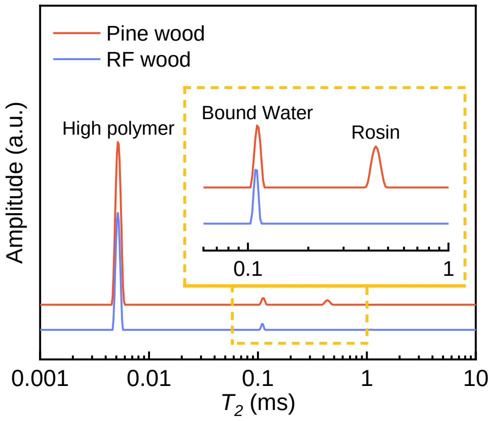

Figure S4: Low field (LF) ^{1}H NMR transverse (T_2) relaxation distribution of pine wood and RF wood.

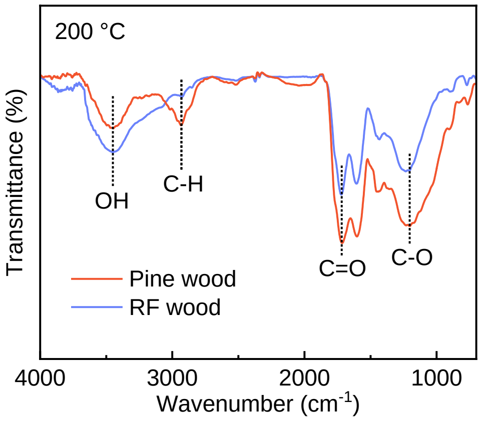

Figure S5: FTIR spectra of pine wood and RF wood after heart treatment at 200 ℃. The intensified C=O stretch and the weakened O−H stretch in Pine HC confirm the occurrence of the esterification reaction. The enhanced C−H stretch is due to the introduction of more methyl and methylene groups by the rosin.

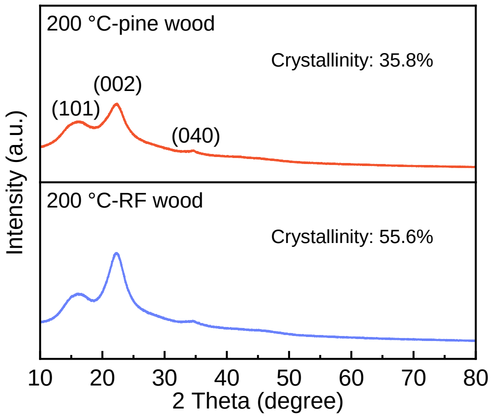

Figure S6: XRD patterns of pine wood and RF wood after heart treatment at 200 ℃. The diffraction peaks of cellulose in both samples did not show significant differences. However, the calculated cellulose crystallinity of pine wood decreased from 55.6% to 35.8% compared to RF wood based on Segal method, suggesting that the esterification process disrupted the intra- and intermolecular hydrogen bonds of cellulose, resulting in lower crystallinity[10]. Furthermore, the reduction in crystallinity can lead to decreased thermal stability of the polymer chains.

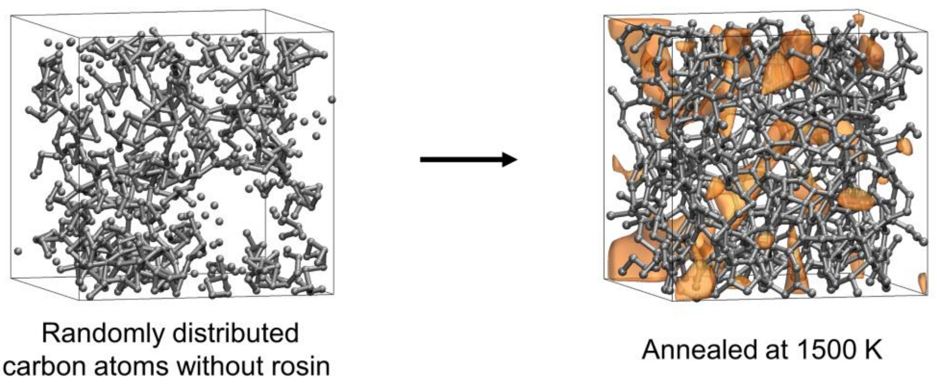

Figure S7: Snapshots of pyrolysis of carbon matrix without rosin. The pores in the carbon matrix are shown in orange. To control the variables, we used the same initial carbon matrix model, only removing rosin. After 1500 K annealing, aromatic cyclic rearrangement occurs between carbon atoms, and the initial large pores disappear, leaving only a few pores.

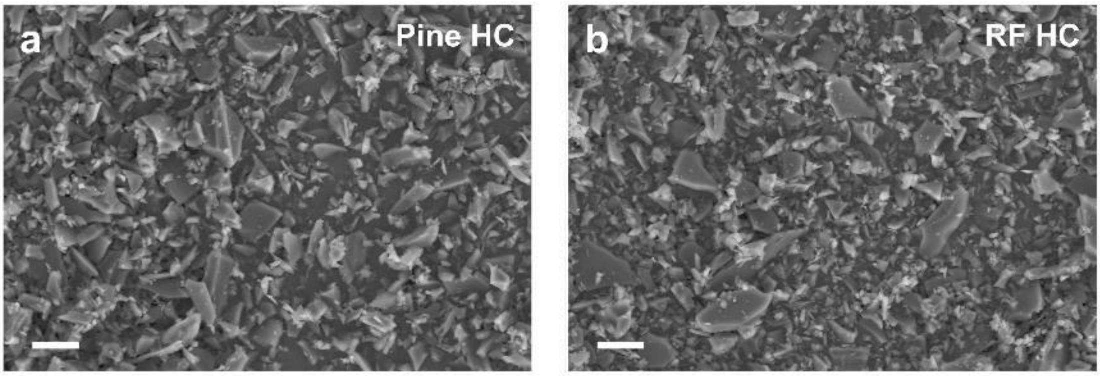

Figure S8: SEM images of Pine HC and RF HC. Scale bars, 10 µm.

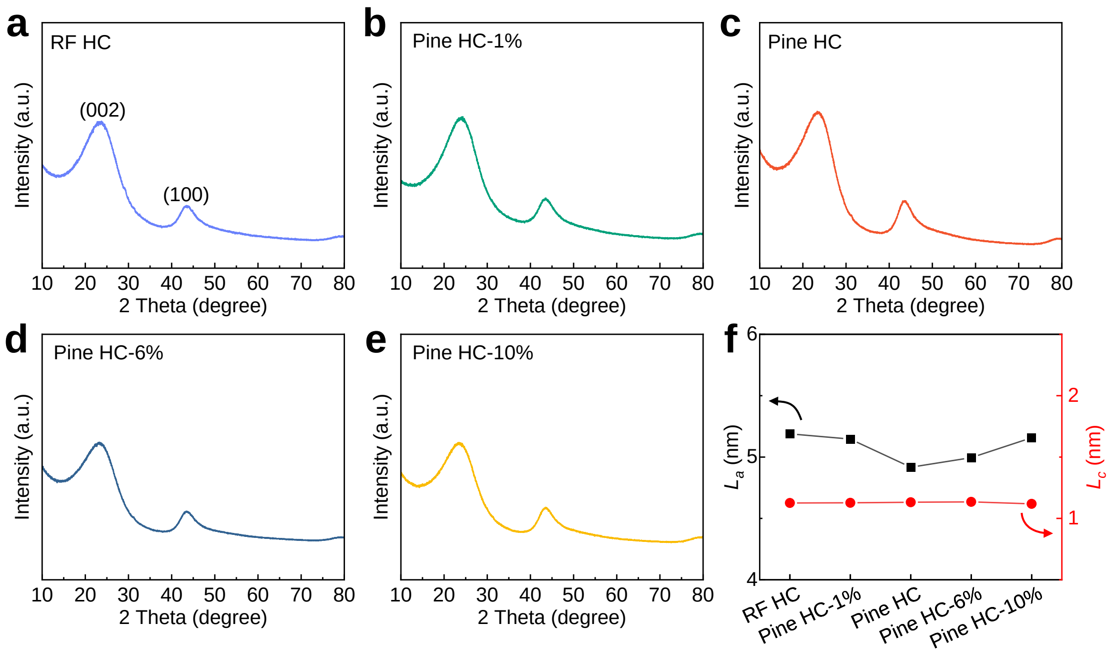

Figure S9: XRD patterns and L_a, L_c parameters of Pine HCs and RF HC.

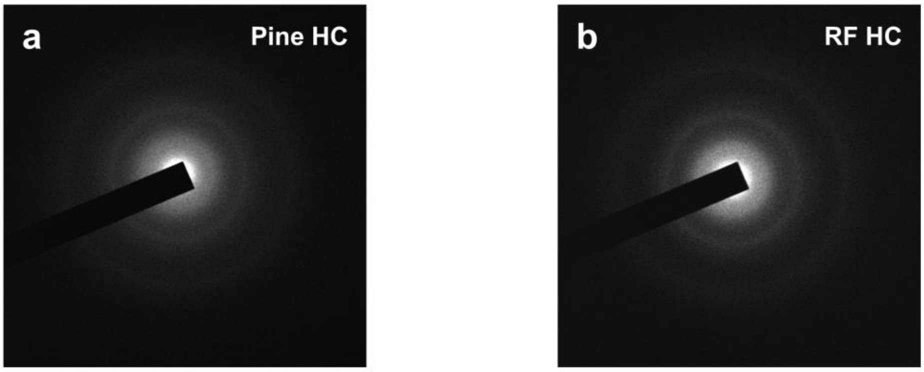

Figure S10: SAED of Pine HC and RF HC.

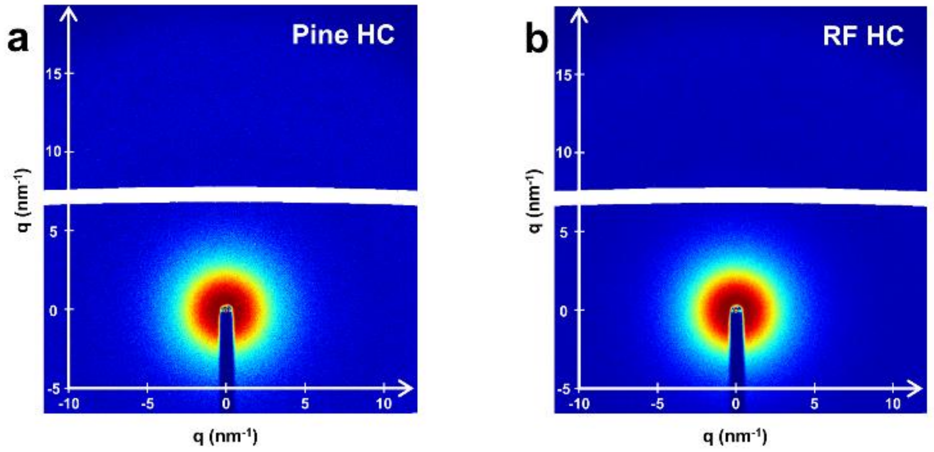

Figure S11: 2D WAXS patterns of Pine HC and RF HC.

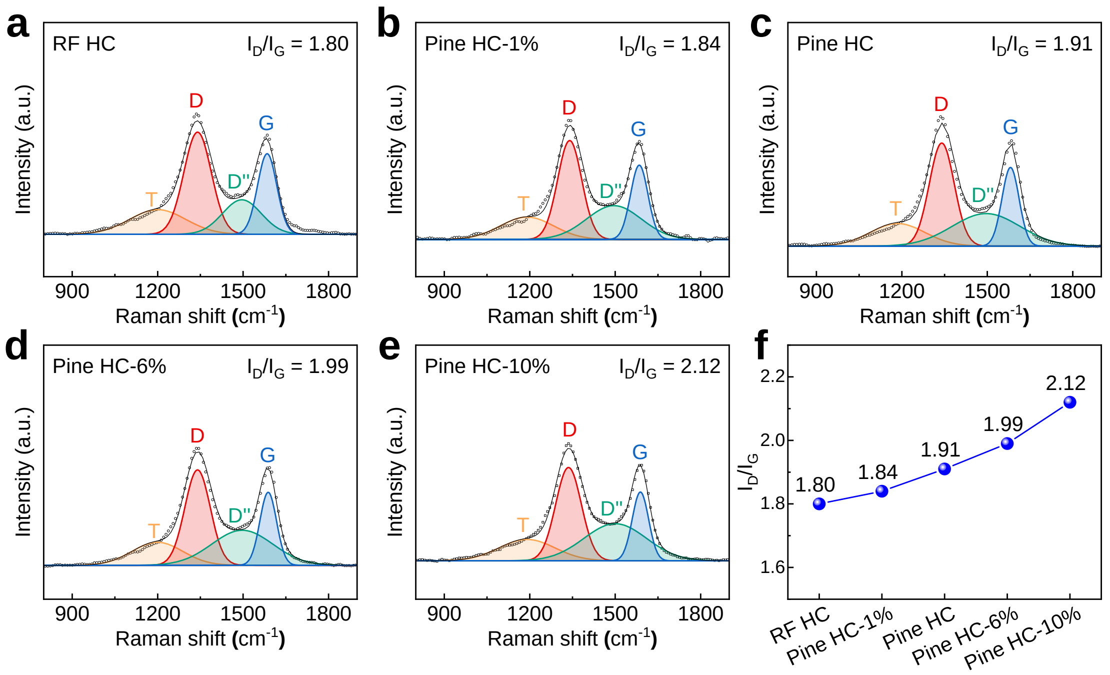

Figure S12: a-f, Raman spectra and I_D/I_G values of Pine HCs and RF HC.

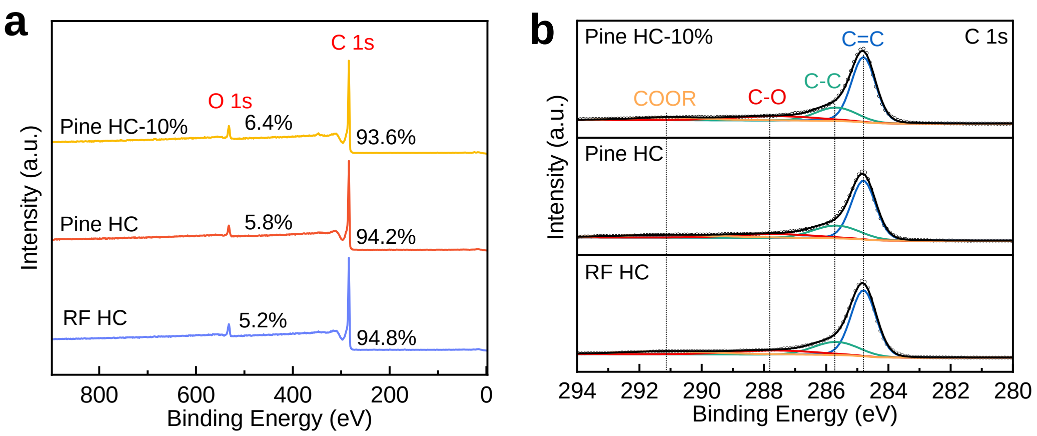

Figure S13: a,**b**, XPS spectra of Pine HC-10%, Pine HC, and RF HC.

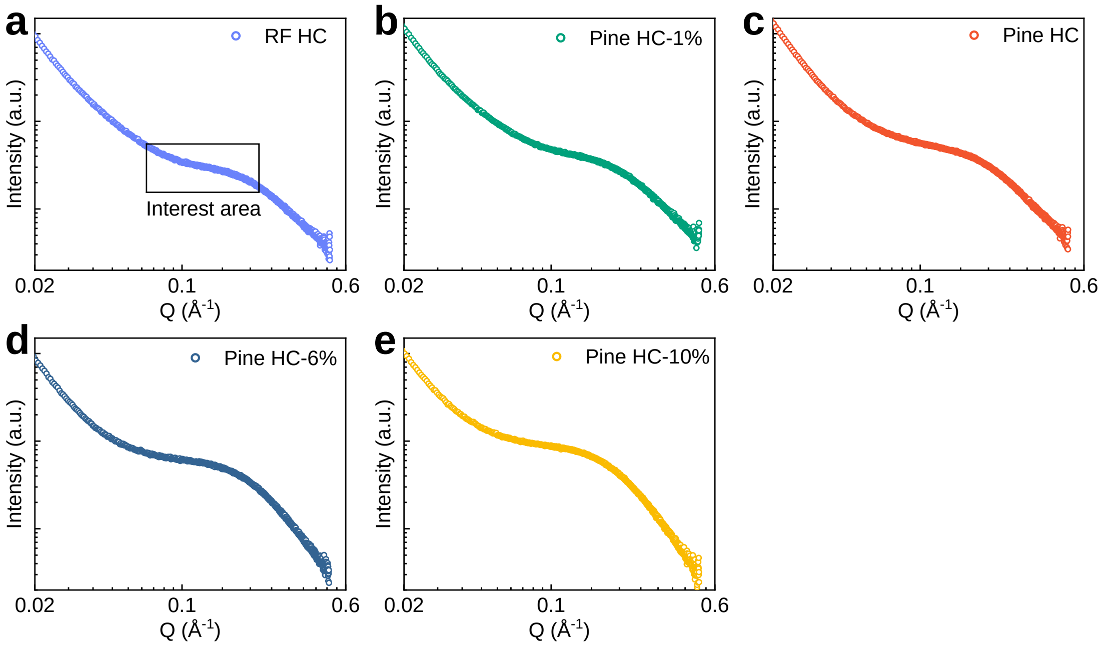

Figure S14: a-d, SAXS profiles of Pine HCs and RF HC.

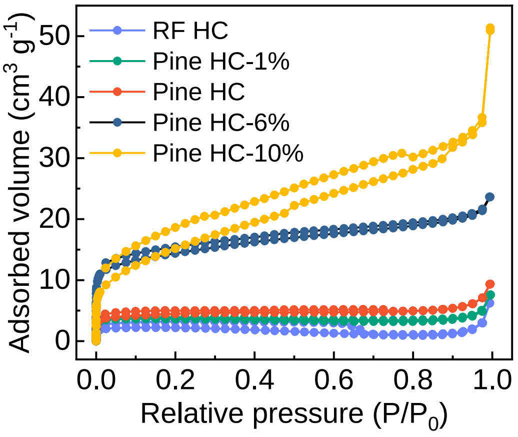

Figure S15: N_2 adsorption/desorption isotherms of Pine HCs and RF HC.

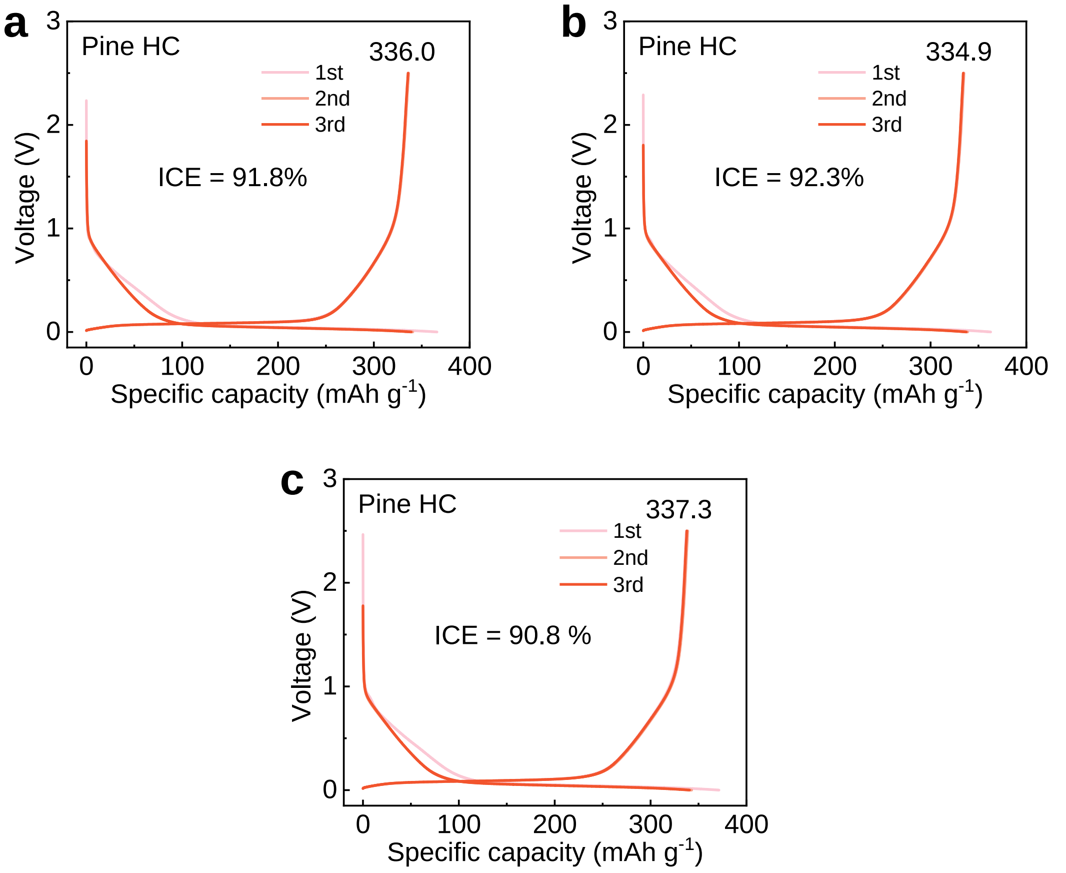

Figure S16: a-c, Charge-discharge profiles of multiple half-cell for Pine HC electrode at 30 mA g^{-1}.

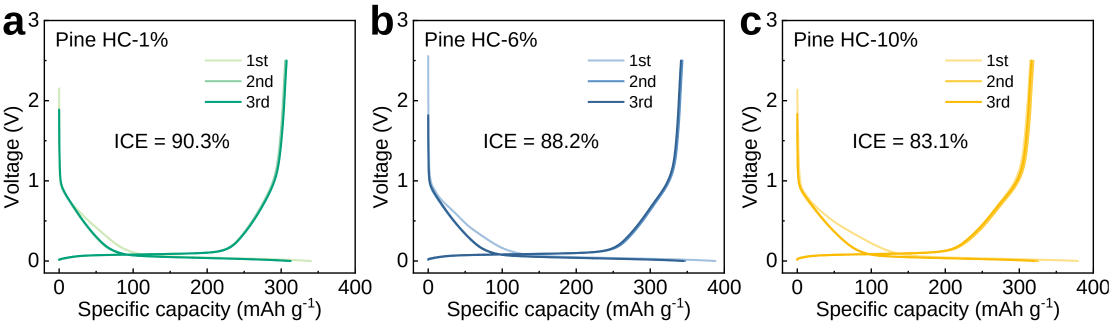

Figure S17: a-c, Galvanostatic charge-discharge profiles of Pine HC-1% (**a**), Pine HC-6% (**b**), and Pine HC-10% (**c**) at 30 mA g^{-1}. As the rosin content increases, the sodium storage capacity and initial Coulombic Efficiency (ICE) of Pine HCs first increase and then decrease.

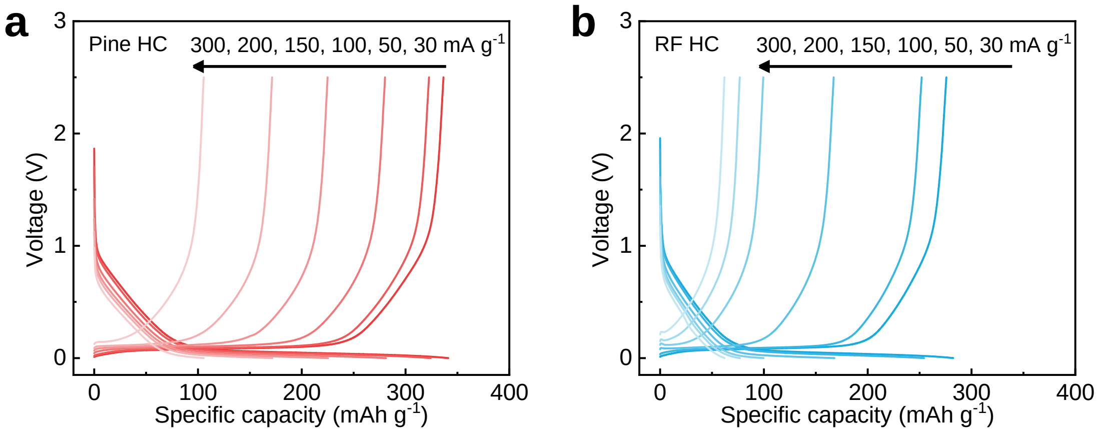

Figure S18: **a**,**b**, Charge-discharge profiles of Pine HC (**a**) and RF HC (**b**) with various current densities ranging from 30 to 300 mA g^{-1}.

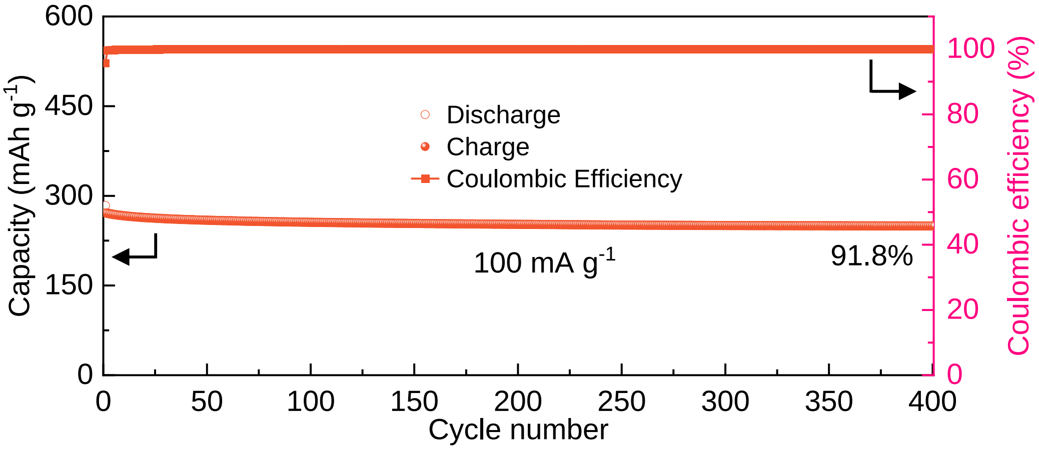

Figure S19: Cycling performance of Pine HC at a current density of 100 mA g^{-1}.

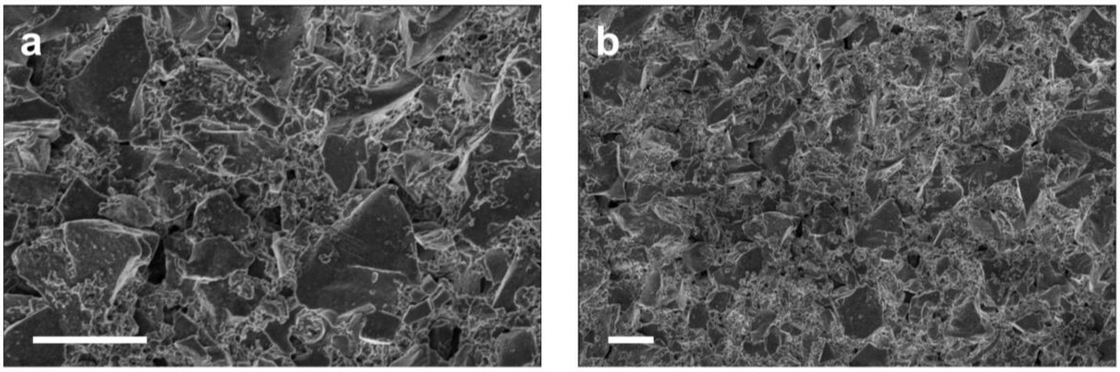

Figure S20: SEM images of Pine HC electrode after 400 cycles at a current density of 100 mA g^{-1}. Scale bars, 2 μm.

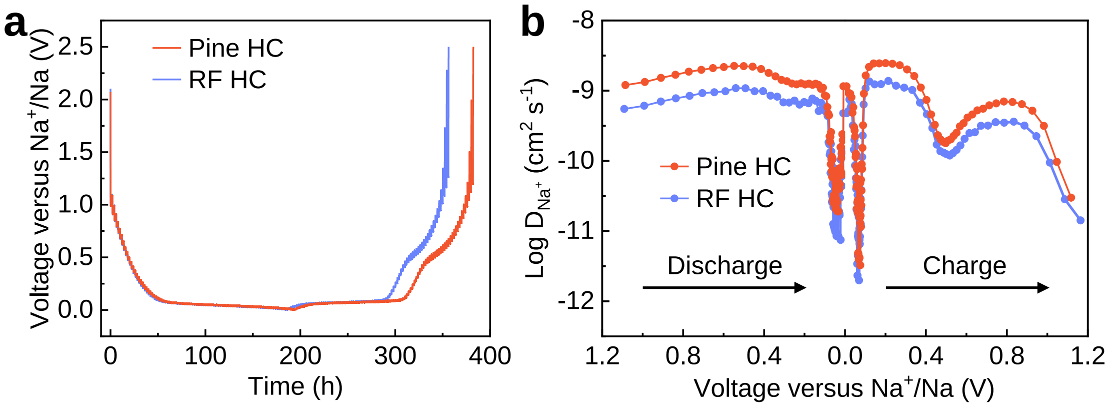

Figure S21: a, The GITT profiles of Pine HC and RF HC. **b,** The corresponding Na^+ ion diffusion coefficients.

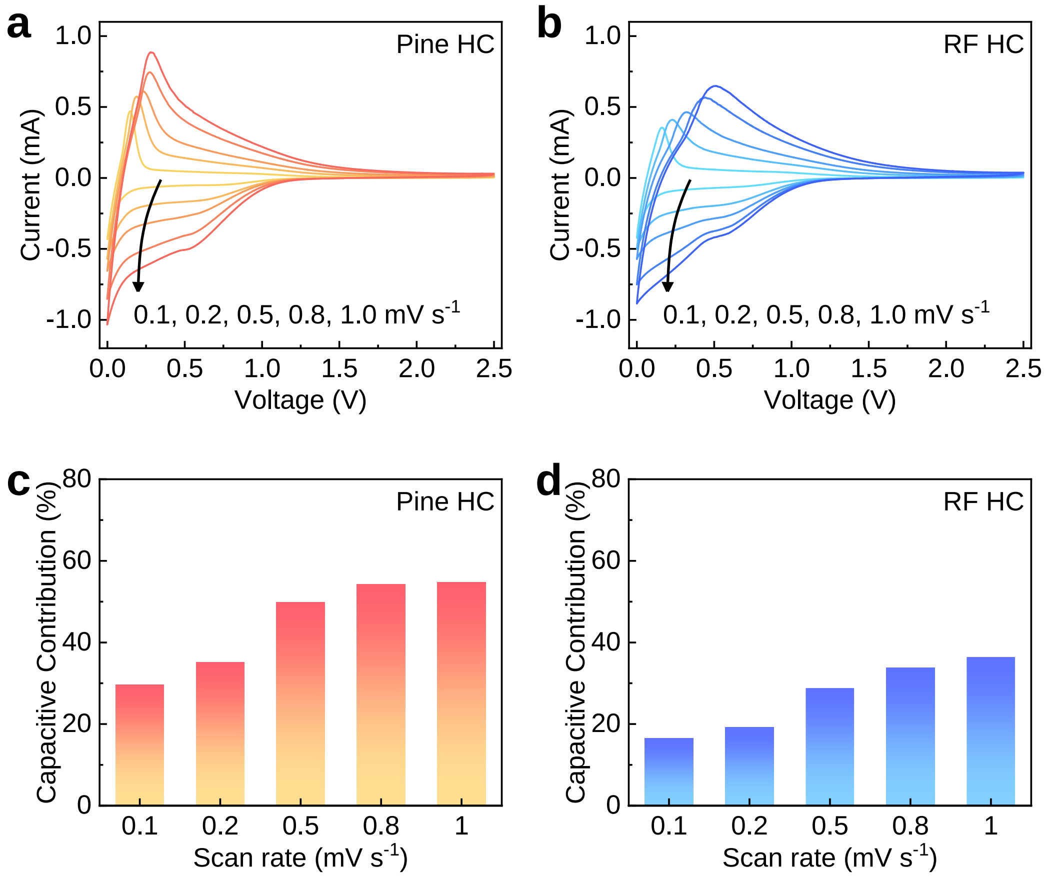

Figure S22: **a**,**b**, CV curves of Pine HC (**a**) and RF HC (**b**) at different scan rates. **c**,**d**, Capacitive contribution of Pine HC (**c**) and RF HC (**d**) at different scan rates.

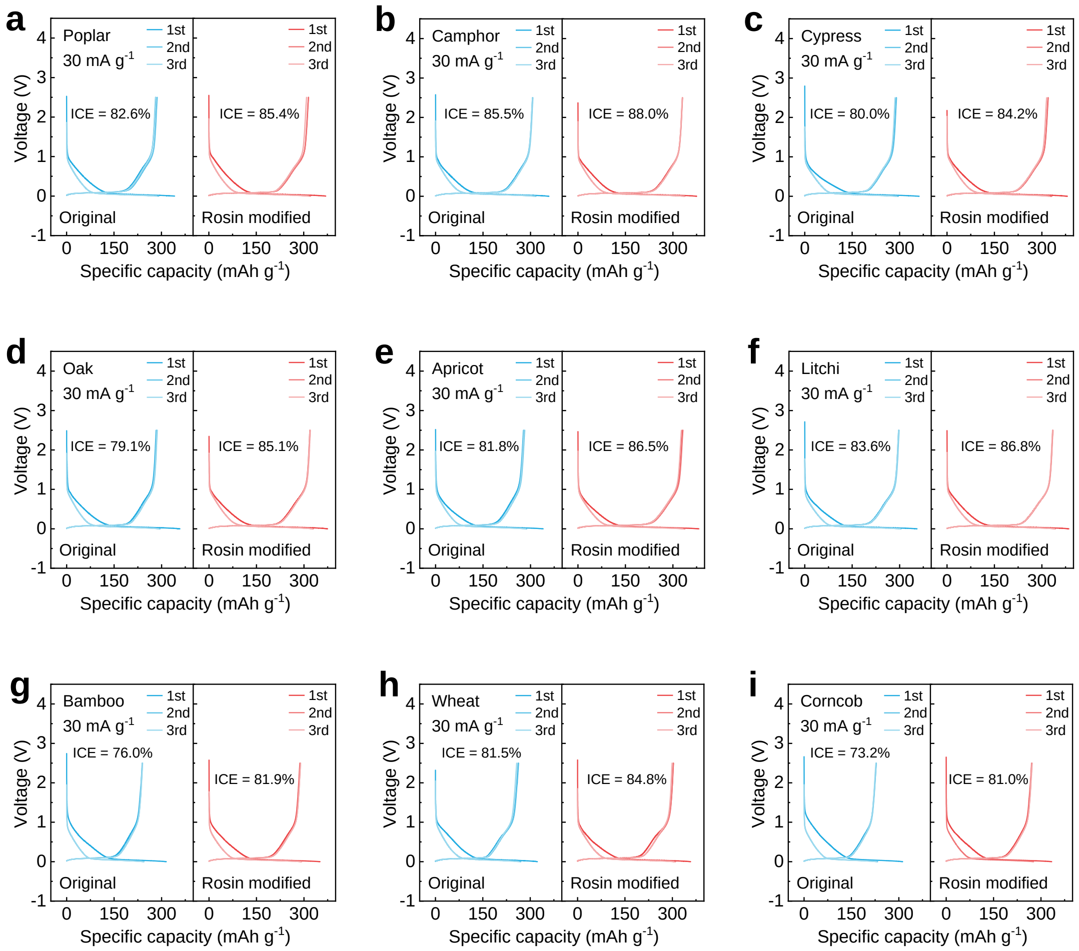

Figure S23: a-h, Charge-discharge profiles of poplar wood (**a**), camphor wood (**b**), cypress wood (**c**), oak wood (**d**), apricot wood (**e**), litchi wood (**f**), bamboo (**g**), wheat straw (**h**), and corncob (**i**)-derived HCs modified with rosin at 30 mA g^{-1}.

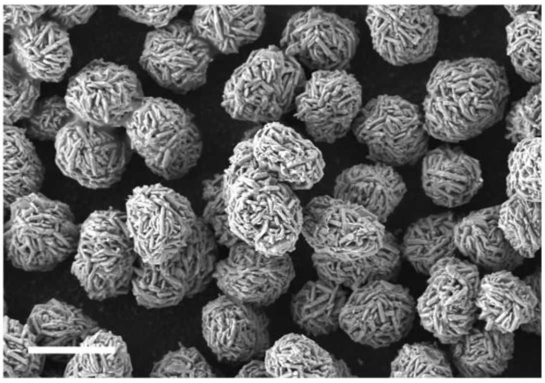

Figure S24: Morphology of NFM111. Scale bar, 5 μm.

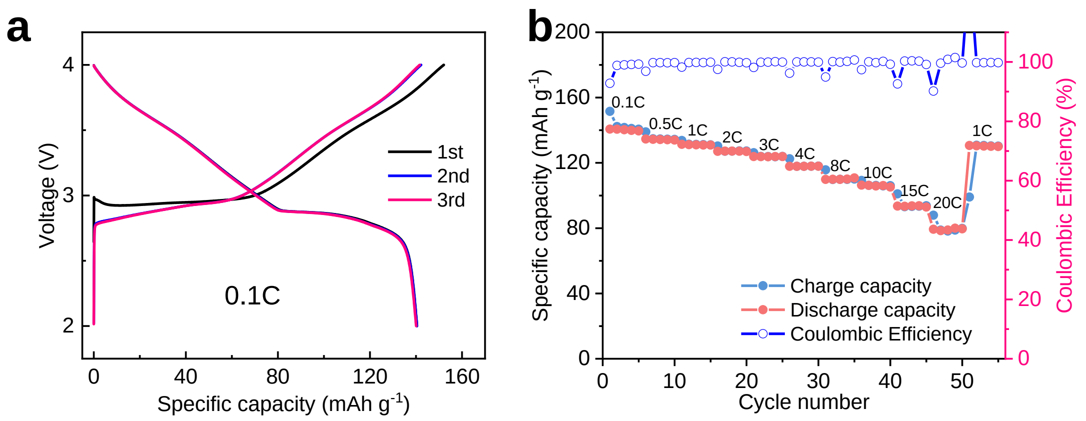

Figure S25: Charge-discharge profiles (**a**) and rate performance (**b**) of NFM111 in half cell.

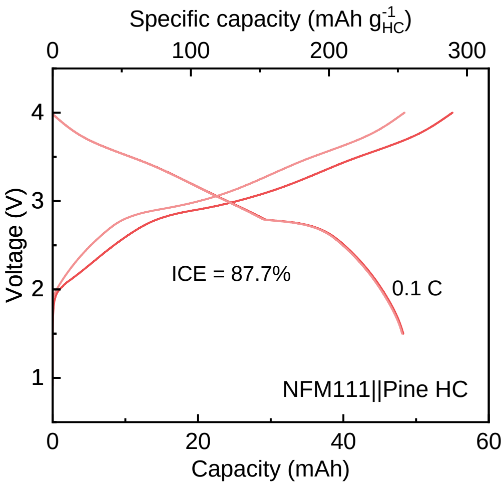

Figure S26: Charge-discharge profiles of NFM111||Pine HC pouch cell during the formation process.

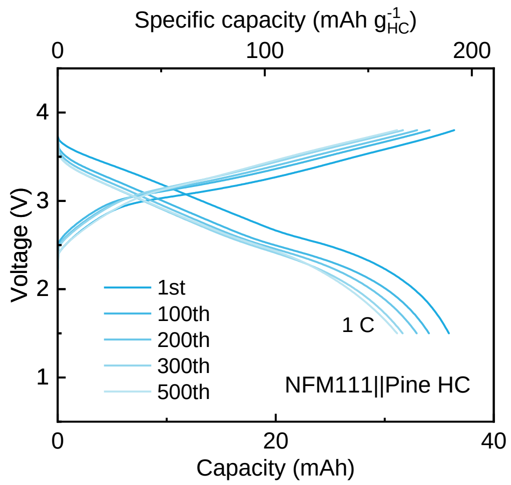

Figure S27: Charge-discharge profiles of NFM111||Pine HC pouch cell at a current rate of 1C.

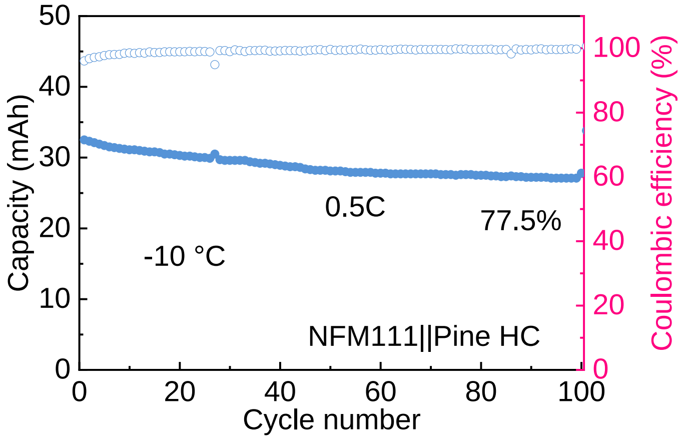

Figure S28: Cycling performance of the NFM111||Pine HC pouch cell at a current rate of 0.5C at -10 ℃.

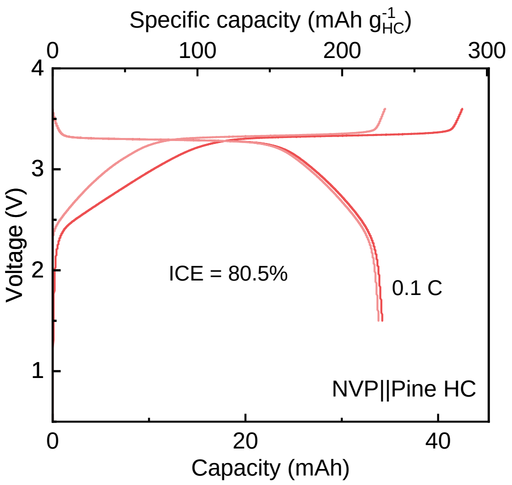

Figure S29: Charge-discharge profiles of NVP||Pine HC pouch cell during the formation process.

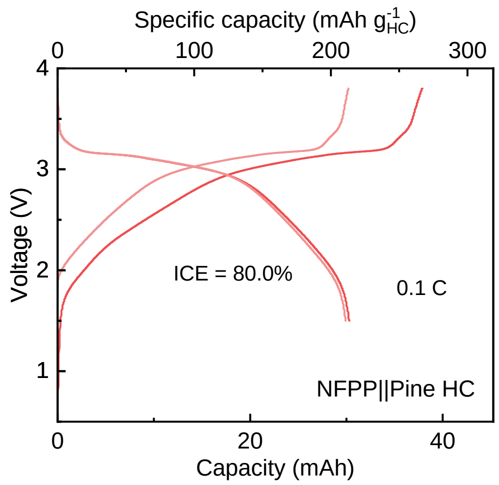

Figure S30: Charge-discharge profiles of NFPP||Pine HC pouch cell during the formation process.

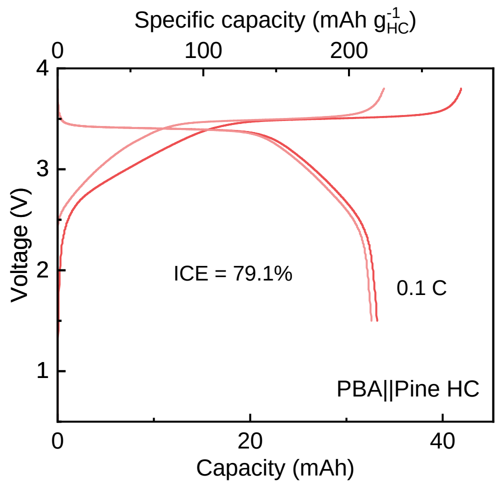

Figure S31: Charge-discharge profiles of PBA||Pine HC pouch cell during the formation process.

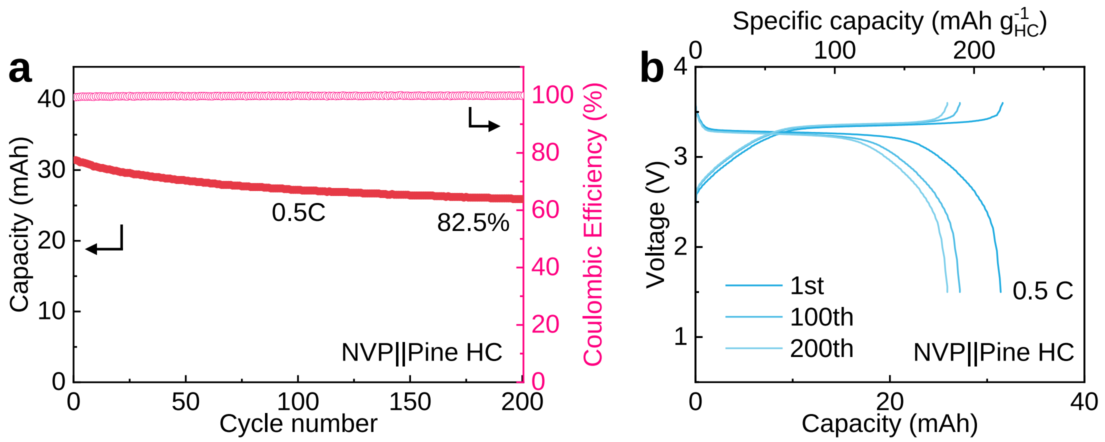

Figure S32: **a**, Cycling performance of the NVP||Pine HC pouch cell at a current rate of 0.5C. **b**, the corresponding charge-discharge profiles.

NVP mass loading: 16.2 mg cm^{-2}; N/P ratio: 1.11; Area: 4.3×5.6 cm^2.

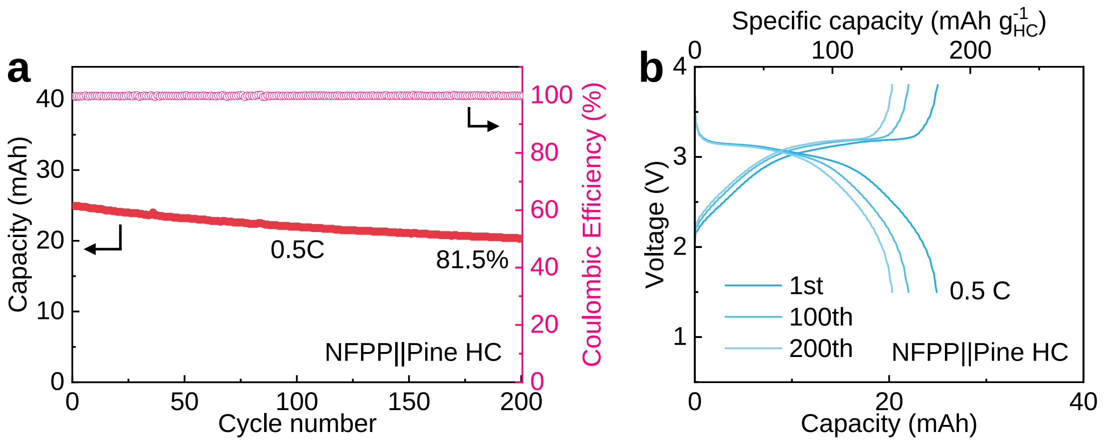

Figure S33: a, Cycling performance of the NFPP||Pine HC pouch cell at a current rate of 0.5C. **b**, the corresponding charge-discharge profiles.

NFPP mass loading: 15.7 mg cm^{-2}; N/P ratio: 1.12; Area: 4.3×5.6 cm^2.

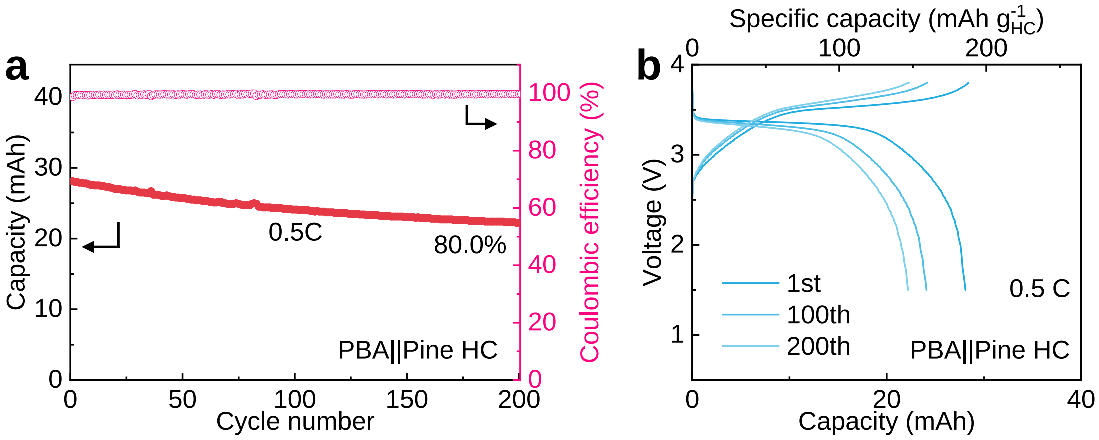

Figure S34: a, Cycling performance of the PBA||Pine HC pouch cell at a current rate of 0.5C. **b**, the corresponding charge-discharge profiles.

PBA mass loading: 10.8 mg cm^{-2}; N/P ratio: 1.19; Area: 4.3×5.6 cm^2.

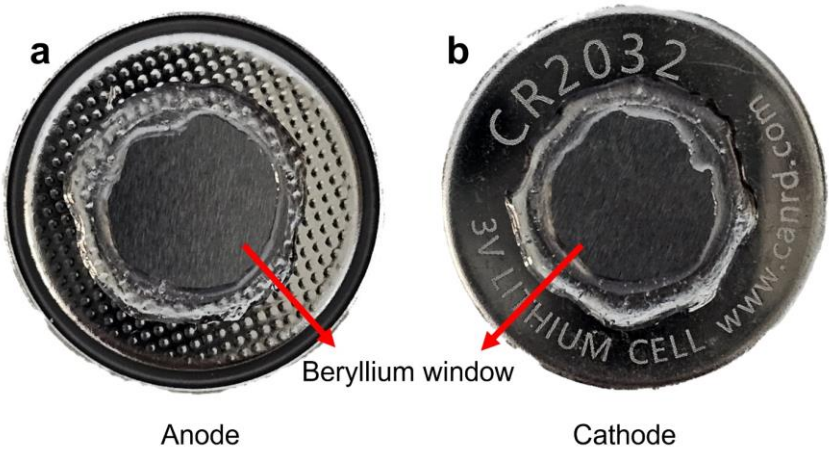

Figure S35: a,**b**, Optical photograph of the in-situ cell. Both the anode (**a**) and cathode (**b**) cases feature a 5 mm diameter window, which is sealed with beryllium window to ensure airtightness while permitting X-rays transmission. A 5 mm diameter hole was made through the sodium metal foil, separator, and stainless steel spacer within the in-situ cell.

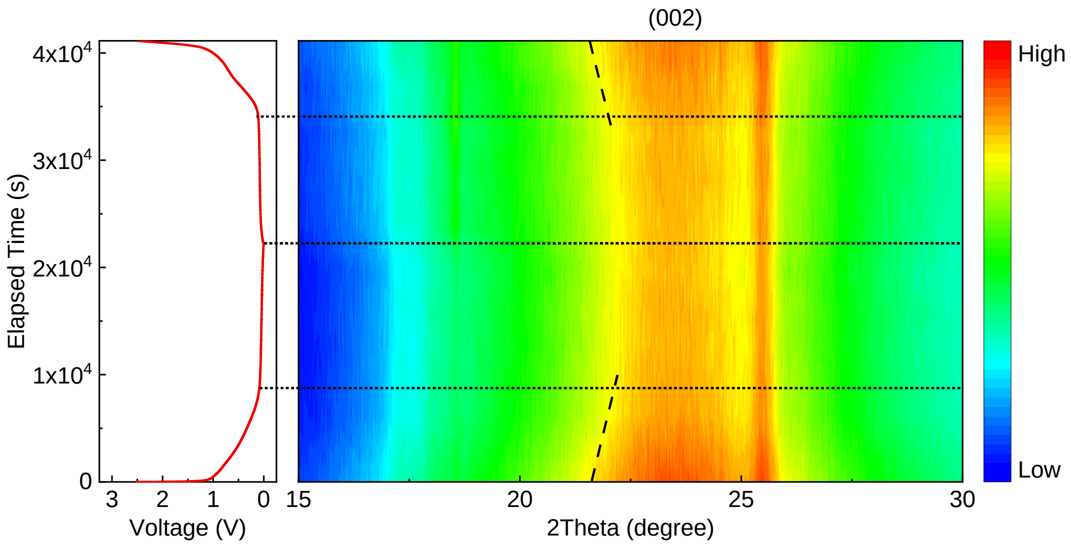

Figure S36: The in-situ XRD patterns of Pine HC electrode during the first charge-discharge process at 30 mA g^{-1}.

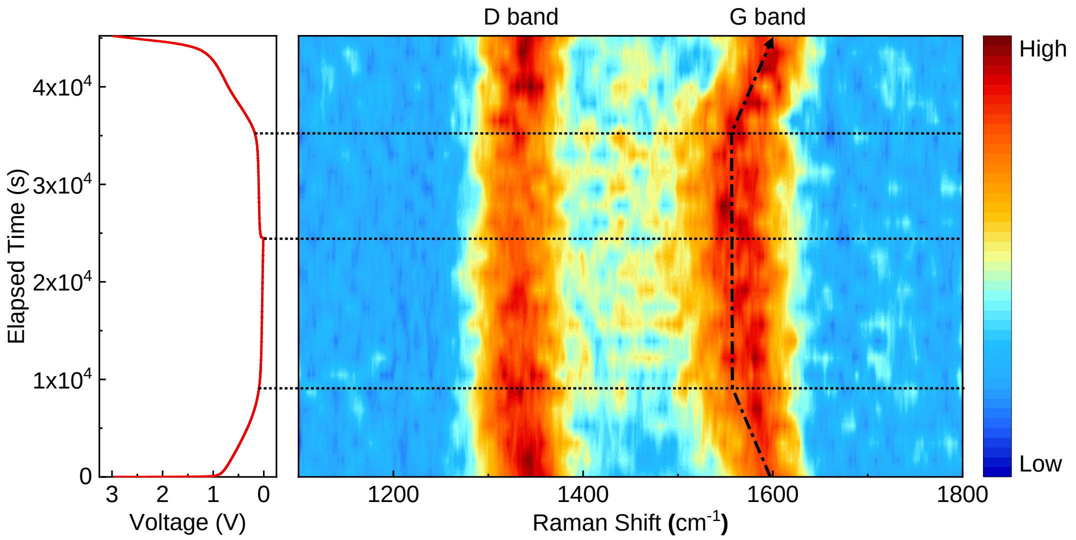

Figure S37: The in-situ Raman spectra of Pine HC electrode during the first charge-discharge process at 30 mA g^{-1}.

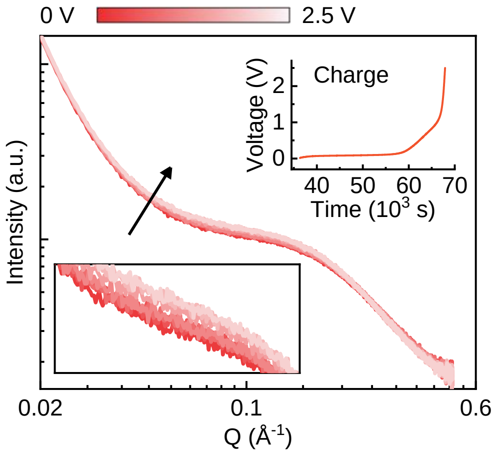

Figure S38: In-situ SAXS profiles of Pine HC electrode during the first desodiation. Curves with various color correspond to different states of charge.

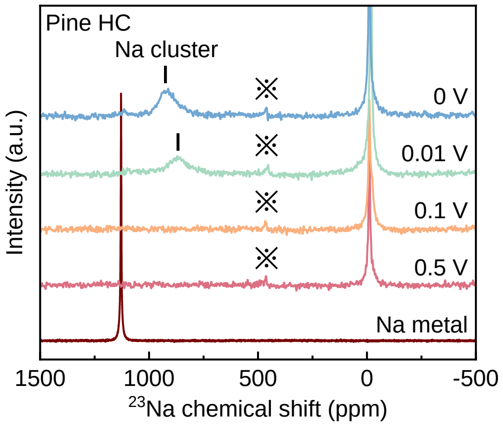

Figure S39: Ex situ ^{23}Na ssNMR spectra of Pine HC electrodes during the first sodiation. Asterisk (※) marks are the spinning sideband.

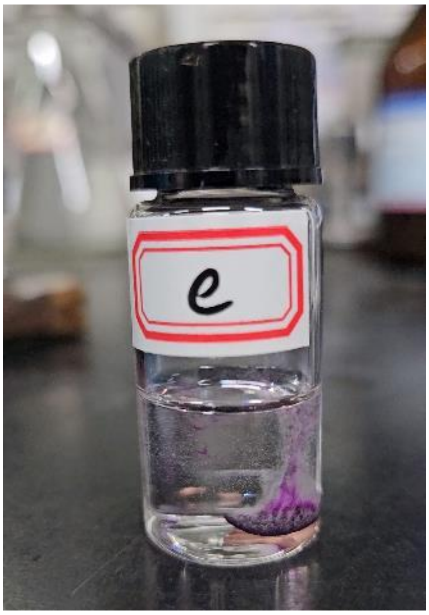

$$nNa^{\delta+} + C_2H_5OH \rightarrow (n-1)Na^{+} + C_2H_5ONa + 1/2H_2 \uparrow \ (0 < \delta < 1)$$

Figure S40: The phenomenon of the reaction between 1% phenolphthalein ethanol solution and sodiated Pine HC and corresponding reaction equation.

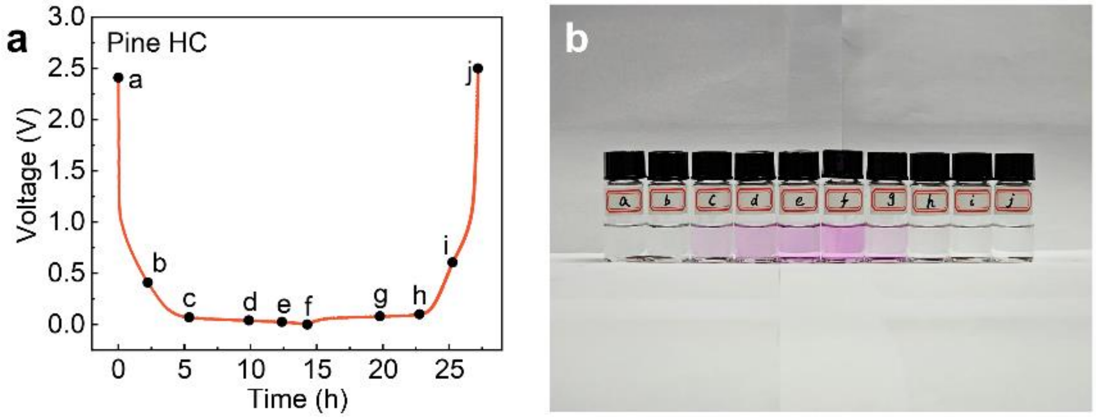

Figure S41: a, The 2nd charge-discharge curves of Pine HC anode. **b,** Optical photo of 1% phenolphthalein ethanol solution after reaction with Pine HC electrode at corresponding voltage.

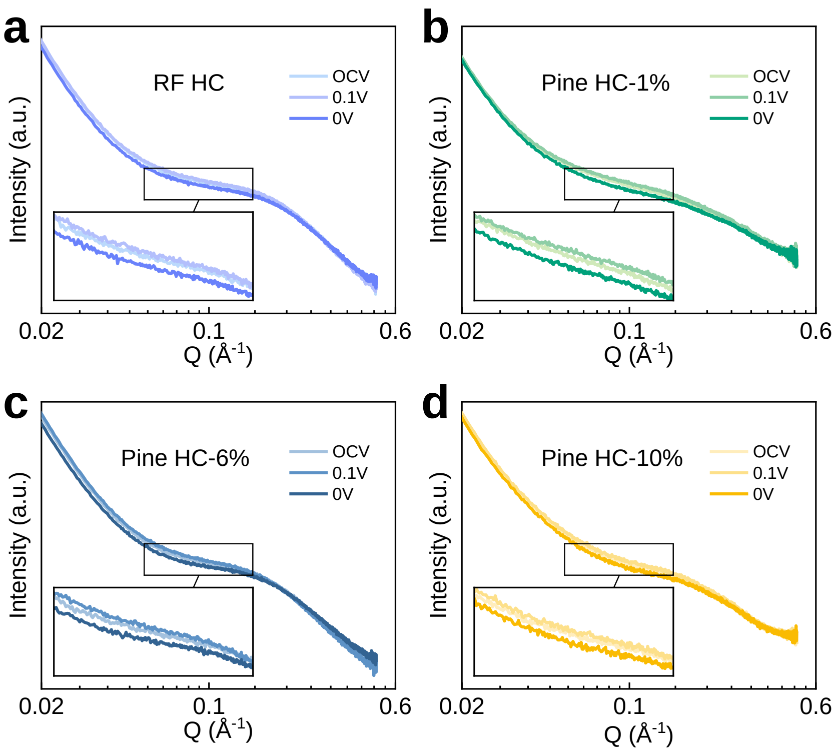

Figure S42: a-d, In-situ SAXS profiles of RF HC (**a**), Pine HC-1% (**b**), Pine HC-6% (**c**), and Pine HC-10% (**d**) electrodes for the first sodiation.

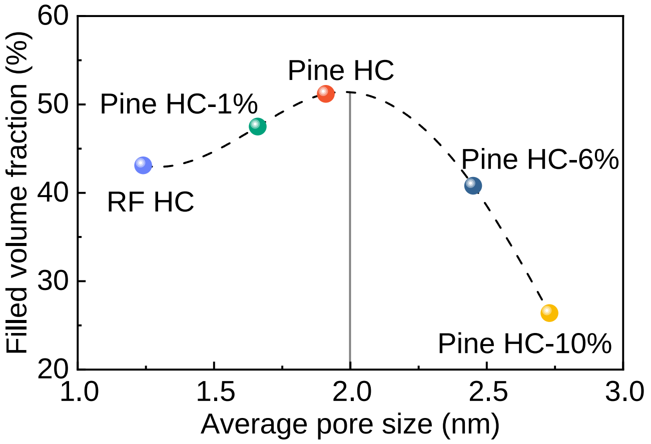

Figure S43: The calculated filled volume fraction from in-situ SAXS experiments.

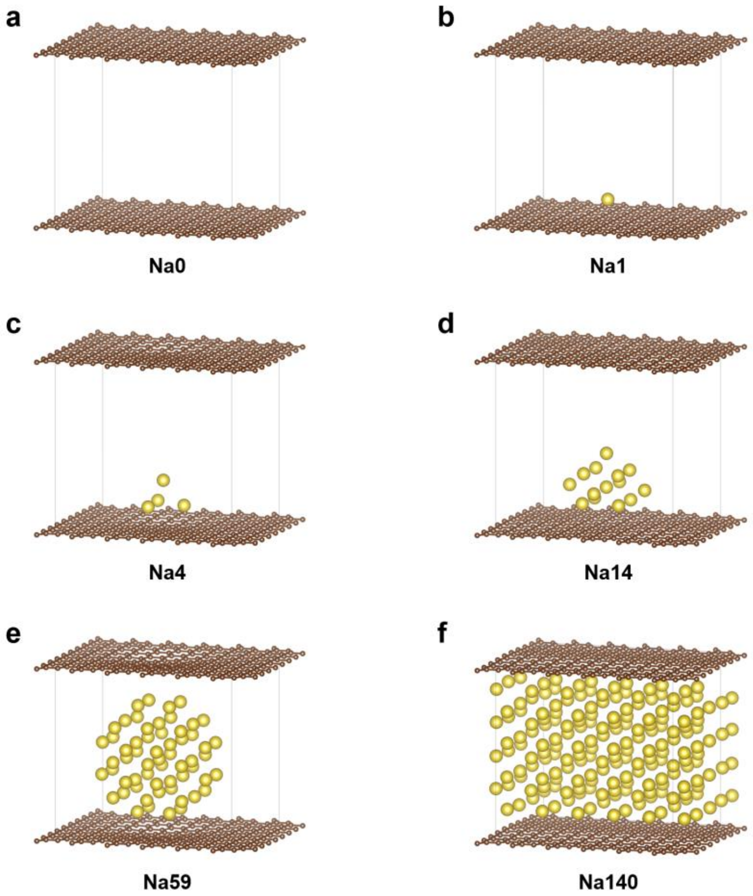

Figure S44: a-f, Structural models of sodium clusters of different sizes adsorbed in closed pores.

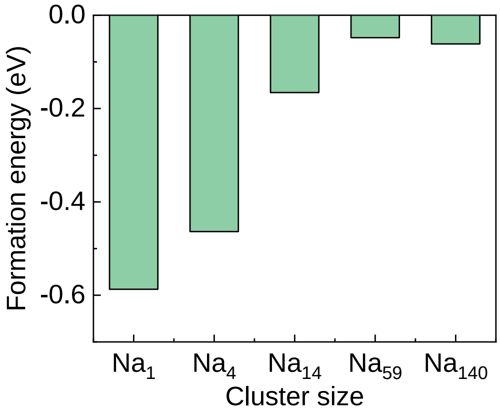

Figure S45: The formation energy of Na clusters (Na_n) with different cluster size.

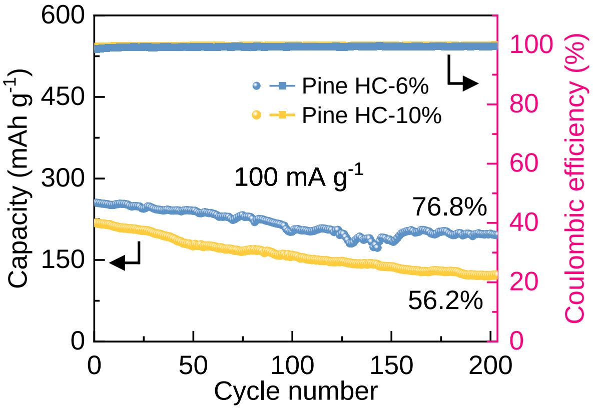

Figure S46: Cycling performance of Pine HC-6% and Pine HC-10% at a current density of 100 mA g^{-1}.

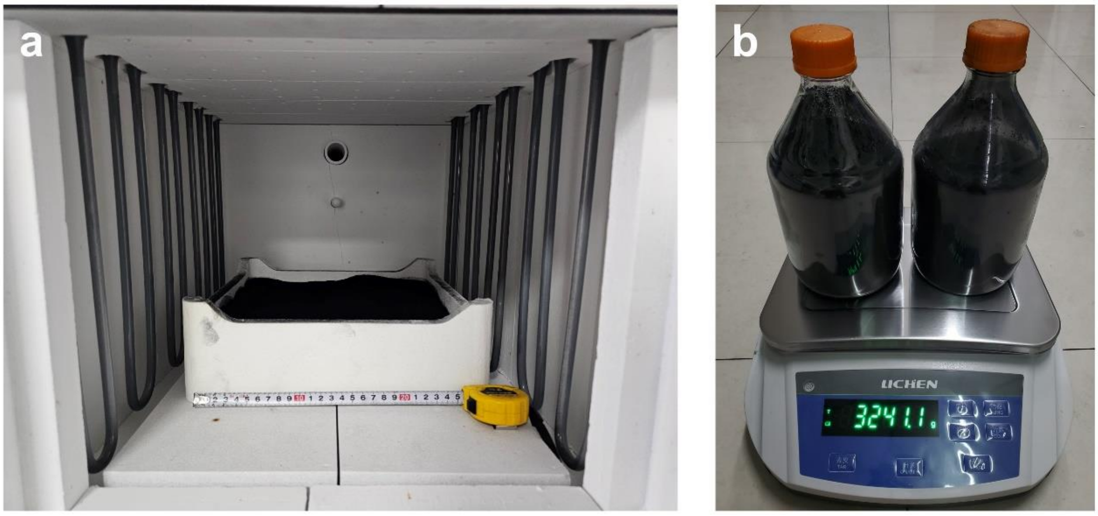

Figure S47: a,**b**, Large-scale preparation of Pine HC (3kg) using pine sawdust as the precursor.

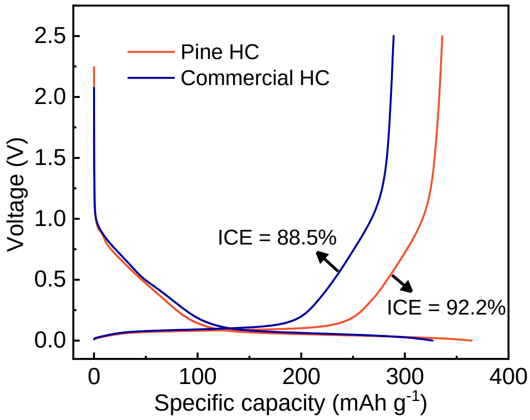

Figure S48: Comparison of charge-discharge profiles of Pine HC and commercial HC in half-cell.

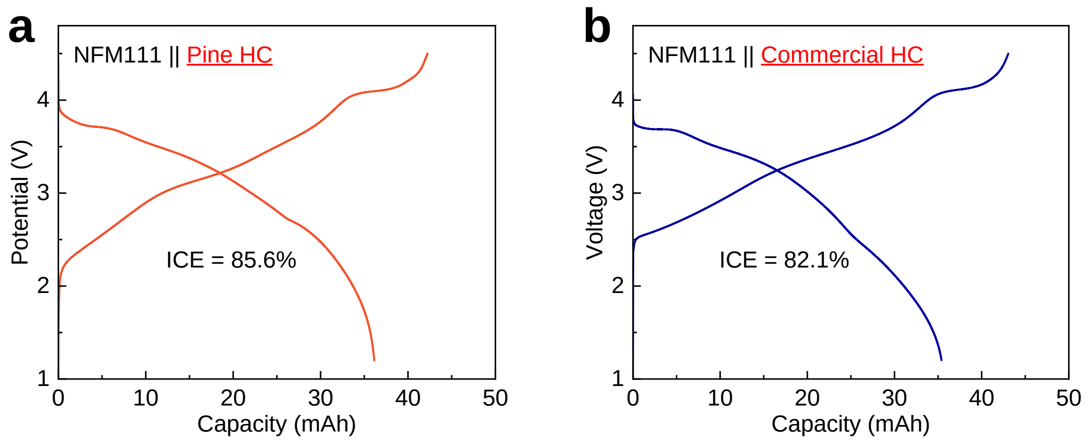

Figure S49: a,**b**, Initial charge-discharge profiles of three-electrode NFM111||Pine HC (**a**) and NFM111||Commercial HC (**b**) pouch cells with an N/P ratio of 1.12.

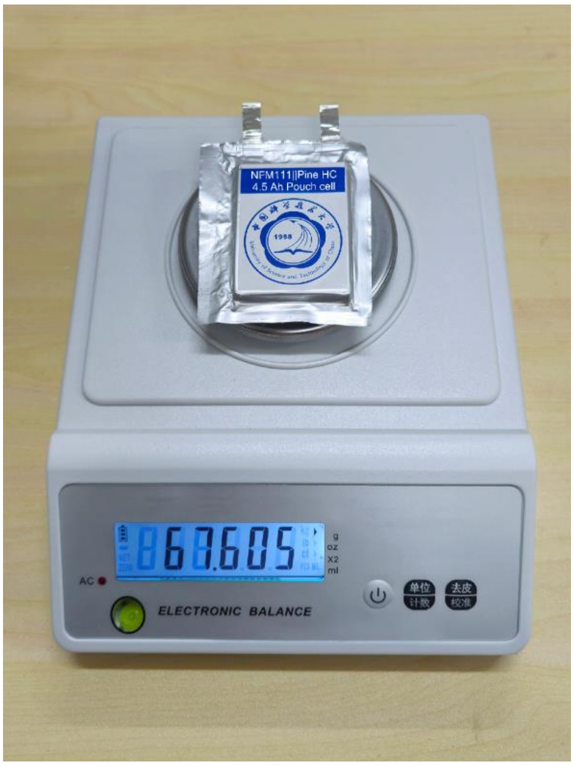

Figure S50: The weight of the 4.5 Ah-laminated NFM111||Pine HC pouch cell.

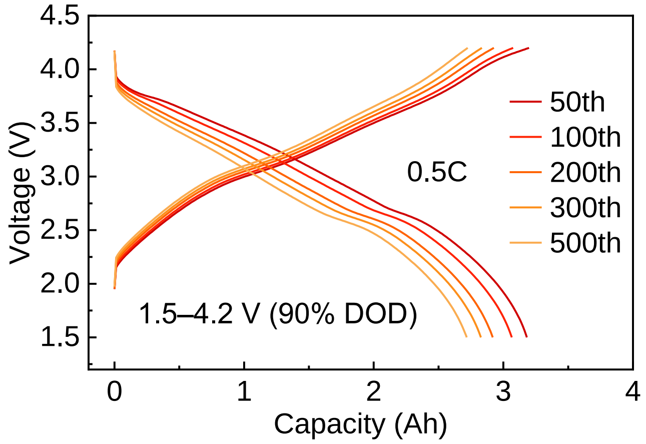

Figure S51: Charge-discharge profiles of the 4.5 Ah-laminated NFM111||Pine HC pouch cell at 90% DOD.

Table S1: Rosin content obtained by Soxhlet extraction.

Sample	Pine wood (g)	Rosin (g)	Rosin content (%)
1	3.5972	0.1140	3.17
2	3.2492	0.1133	3.49
3	3.4314	0.1118	3.26

Table S2: ^{13}C chemical shift assignments of pine wood and RF wood.

Chemical shift (ppm)	Carbon group	Belonging*
180−170	C=O	R, H
158−117	Aromatic	L
110−58	C1−C6	C
56	CH_3O	L
50−12	CH_3, CH_2	R

*Rosin, cellulose, hemicellulose, and lignin were labeled as R, C, H, and L, respectively.

Table S3: The functional groups assignments of pine wood and RF wood.

Wavenumber (cm^{-1})	Functional groups	Belonging
3564	O−H stretching	H_2O
2944	C−H stretching	CH_4
2340	C=O stretching	CO_2
2180	C=O stretching	CO
1742	C=O	Aldehydes/acid
1108	C−O−C/C−C	Ethers/alkanes

Table S4: Pore parameters of carbon matrix with/without rosin after pyrolysis by AIMD simulations.

Samples	Carbon matrix with rosin	Pure carbon matrix
Free volume (Å^3)	1829.95	1287.54
Free volume ratio	22.87%	16.09%
Surface area (Å^3)	3969.95	3671.18

Table S5: Structure parameters (d_{002}, d_{100}, L_a, L_c, and n) of RF HC and Pine HC samples based on XRD patterns.

Sample	d_{002} (nm)	d_{100} (nm)	L_a (nm)	L_c (nm)	n=L_c/d_{002}+1
Pine HC-10%	0.377	0.208	5.157	1.119	3.968
Pine HC-6%	0.380	0.208	4.995	1.135	3.987
Pine HC	0.378	0.208	4.917	1.132	3.995
Pine HC-1%	0.376	0.207	5.146	1.127	3.997
RF HC	0.373	0.206	5.189	1.126	4.019

Table S6: SAXS fitting parameters of Pine HC and RF HC samples based on the Teubner-Strey model.

Sample	ρ_s (g cm^{-3})	ΔSLD (*10^{-6} Å^{-2})	d (nm)	ξ (nm)	D (nm)	S_{SAXS} (m^2 g^{-1})
Pine HC-10%	2.41	20.5	11.58	0.47	2.73	4392
Pine HC-6%	2.42	20.6	8.75	0.45	2.45	3650
Pine HC	2.40	20.4	5.40	0.46	1.91	2916
Pine HC-1%	2.36	20.1	4.73	0.48	1.66	2333
RF HC	2.32	19.7	4.2	0.53	1.24	1528

Table S7: Pore structure parameters of RF HC and Pine HC samples based on nitrogen adsorption measurement and true density test.

Sample	S_{BET} ($m^2\ g^{-1}$)	Open pore volume ($cm^3\ g^{-1}$)	True density ($g\ cm^{-3}$)	Closed pore volume ($cm^3\ g^{-1}$)	Total pore volume ($cm^3\ g^{-1}$)
Pine HC-10%	52.4	0.051	1.81	0.110	0.161
Pine HC-6%	30.3	0.029	1.90	0.084	0.113
Pine HC	16.7	0.013	1.97	0.065	0.078
Pine HC-1%	14.9	0.012	2.02	0.053	0.065
RF HC	9.0	0.009	2.09	0.036	0.045

Table S8: Comparison of the specific capacity and ICE of previously reported high-performance HC anodes with Pine HC.

Sample	Specific capacity (mAh g^{-1})	ICE	Reference
CHC	341	68.0%	[11]
CHC-0.25	325	68.0%	[12]
LCS-73	356	82.8%	[13]
HTCNCC1500	314	90.4%	[14]
H-TPGC	298	90.5%	[15]
G1500	345	86.9%	[16]
PCLC-1	312	85.3%	[17]
CAC1300	308	82.3%	[18]
HC-1300	350	84.1%	[19]
HC-1300-P1.10	328	72.0%	[20]
HCK-10	332	68.0%	[21]
M11005	299	88.9%	[22]
MPC-1000	318	71.2%	[23]
F-CHC	335	87.2%	[24]
RC-90	524	82.3%	[25]
N-HGCF	426.4	20.5%	[26]
Pine HC	336	92.2%	This work

Table S9: Comparison of the electrochemical performance* of other biomass-derived HCs before and after rosin modification.

Biomass	Basic			Rosin-modified		
	Slope capacity (mAh g^{-1})	Plateau capacity (mAh g^{-1})	ICE	Slope capacity (mAh g^{-1})	Plateau capacity (mAh g^{-1})	ICE
Poplar wood	78.5	213.0	82.6%	86.7	234.7	85.4%
Camphor wood	84.9	232.1	85.5%	86.1	251.3	88.0%
Cypress wood	77.7	218.0	80.0%	93.5	237.9	84.2%
Oak wood	80.3	214.9	79.1%	94.4	235.1	85.1%
Apricot wood	77.9	210.0	81.8%	90.2	248.0	86.5%
Litchi wood	76.3	228.2	83.6%	91.5	251.3	86.8%
Bamboo	68.8	176.1	76.0%	72.4	219.9	81.9%
Wheat straw	80.8	189.3	81.5%	83.8	226.2	84.8%
Corncob	67.99	164.66	73.2%	72.7	203.78	81.0%

*Current density: 30 mA g^{-1}.

Table S10: Cost accounting for large-scale preparation of Pine HC using pine sawdust as raw material.

	Pine HC	Commercial HC (Kuraray Type 2)
Raw material	Pine sawdust	\
Precursor price range[#] (\$ kg^{-1})	0.26−0.38	\
Average yield	25%	\
Production fixed cost range[*] (\$ kg^{-1})	2.0−2.5	\
Total cost (\$ kg^{-1})	3.04−4.02	\
Average cost (\$ kg^{-1})	3.53	\
Price (\$ kg^{-1})	\	27.5

[#]The precursor price was consulted at Huadong Timber Market (Jinhua, Zhejiang).

[*]The calculation of the production fixed cost for Pine HC is based on techno-economics studies that producing activated carbon from biomass materials.[27-29] These studies estimated fixed production costs excluding raw material costs to be between \$1.50-2.50 kg^{-1}. Taking inflation and energy prices differences into account, the fixed production costs for Pine HC were assumed to be \$2.0-2.5 kg^{-1}.

Table S11: Comparison of electrochemical performance between Pine HC and commercial HC.

	Pine HC	Commercial HC (Kuraray Type 2)
**Half cell:**		
Initial Coulombic efficiency	92.2%	88.5%
Reversible capacity (mAh g^{-1})	336.1	289.2
Average voltage (V)	0.14	0.19
**Pouch type full cell:**		
Initial Coulombic efficiency	85.6%	82.1%
Reversible capacity (mAh g^{-1})	272.1	229.6
Average discharge voltage (V)	3.09	2.98
Energy density (W h kg^{-1})	315.8	283.4

Table S12: The detailed parameters of laminated NFM111||Pine HC pouch cell with an energy density of 202 W h kg^{-1}.

Parameter	NFM111||Pine HC pouch cell
Specific capacity (Pine HC)	365 mAh g^{-1}
Anode mass loading (94 wt%)	7.64 mg cm^{-2}
Area capacity (single face)	2.79 mAh cm^{-2}
Active area (40 folds, double face)	2088.0 cm^2 (4.5×5.8 cm^2)
Specific capacity (NFM111)	200 mAh g^{-1}
Cathode mass loading (95 wt%)	13.71 mg cm^{-2}
Area capacity (single face)	2.74 mAh cm^{-2}
Active area (39 folds, double face)	1878.2 cm^2 (4.3×5.6 cm^2)
N/P ratio	1.02
Current collector (9 μm Al foil)	5.12 g
Separator (Liaoyuan Hongtu, 12 μm)	1.53 g
E/C ratio	3.47 g Ah^{-1}
Package + tabs	1.67 g
Cell weight	67.61 g
Cell capacity	4.45 Ah
Cell energy	13.66 Wh
Energy density	202 Wh kg^{-1}

References

[1] Y. Wang, Z. Li, X. Li, Z. F. Ma, L. Li, *Adv. Energy Mater.* **2024**, 14, 2304512.

[2] D. Saurel, J. Segalini, M. Jauregui, A. Pendashteh, B. Daffos, P. Simon, M. Casas-Cabanas, *Energy Stor. Mater.* **2019**, 21, 162.

[3] L. Kitsu Iglesias, E. N. Antonio, T. D. Martinez, L. Zhang, Z. Zhuo, S. J. Weigand, J. Guo, M. F. Toney, *Adv. Energy Mater.* **2023**, 13, 2302171.

[4] Y. Q. Li, Y. X. Lu, Q. S. Meng, A. C. S. Jensen, Q. Q. Zhang, Q. H. Zhang, Y. X. Tong, Y. R. Qi, L. Gu, M. M. Titirici, Y. S. Hu, *Adv. Energy Mater.* **2019**, 9, 1902852.

[5] L. Martínez, R. Andrade, E. G. Birgin, J. M. Martínez, *Journal of Computational Chemistry* **2009**, 30, 2157.

[6] T. D. Kühne, M. Iannuzzi, M. Del Ben, V. V. Rybkin, P. Seewald, F. Stein, T. Laino, R. Z. Khaliullin, O. Schütt, F. Schiffmann, *J. Chem. Phys.* **2020**, 152, 194103.

[7] S. Goedecker, M. Teter, J. Hutter, *Physical Review B* **1996**, 54, 1703.

[8] J. P. Perdew, K. Burke, M. Ernzerhof, *Phys. Rev. Lett.* **1996**, 77, 3865.

[9] G. Bussi, D. Donadio, M. Parrinello, *J. Chem. Phys.* **2007**, 126, 014101.

[10] S. Nam, A. D. French, B. D. Condon, M. Concha, *Carbohydr. Polym.* **2016**, 135, 1.

[11] Z. Tang, S. Zhou, P. Wu, H. Wang, Y. Huang, Y. Zhang, D. Sun, Y. Tang, H. Wang, *Chem. Eng. J.* **2022**, 441, 135899.

[12] S. Guo, Y. Chen, L. Tong, Y. Cao, H. Jiao, Z. long, X. Qiu, *Electrochim. Acta* **2022**, 410, 140017.

[13] H. Chen, N. Sun, Q. Zhu, R. A. Soomro, B. Xu, *Adv. Sci.* **2022**, 9, 2200023.

[14] F. Xie, Z. Xu, Z. Guo, A. C. S. Jensen, J. Feng, H. Luo, F. Ding, Y. Lu, Y. S. Hu, M. M. Titirici, *Carbon Energy* **2022**, 4, 914.

[15] X. X. He, W. H. Lai, Y. Liang, J. H. Zhao, Z. Yang, J. Peng, X. H. Liu, Y. X. Wang, Y. Qiao, L. Li, X. Wu, S. L. Chou, *Adv. Mater.* **2023**, 35, e2302613.

[16] Z. Guo, Z. Xu, F. Xie, J. Jiang, K. Zheng, S. Alabidun, M. Crespo-Ribadeneyra, Y. S. Hu, H. Au, M. M. Titirici, *Adv. Mater.* **2023**, 35, e2304091.

[17] H. Chen, N. Sun, Y. Wang, R. A. Soomro, B. Xu, *Energy Stor. Mater.* **2023**, 56, 532.

[18] K. F. Wang, F. Sun, H. Wang, D. Y. Wu, Y. X. Chao, J. H. Gao, G. B. Zhao, *Adv. Funct. Mater.* **2022**, 32, 2203725.

[19] T. Xu, X. Qiu, X. Zhang, Y. Xia, *Chem. Eng. J.* **2023**, 452, 139514.

[20] S. Alvin, C. Chandra, J. Kim, *Chem. Eng. J.* **2020**, 391, 123576.

[21] R. Dong, F. Wu, Y. Bai, Q. Li, X. Yu, Y. Li, Q. Ni, C. Wu, *Energy Mater. Adv.* **2022**, 2022, 9896218.

[22] Y. Zhen, Y. Chen, F. Li, Z. Guo, Z. Hong, M. M. Titirici, *Proc. Natl. Acad. Sci. U. S. A.* **2021**, 118, e2111119118.

[23] J. L. Xia, D. Yan, L. P. Guo, X. L. Dong, W. C. Li, A. H. Lu, *Adv. Mater.* **2020**, 32, e2000447.

[24] Y. Chen, F. Li, Z. Guo, Z. Song, Y. Lin, W. Lin, L. Zheng, Z. Huang, Z. Hong, M.-M. Titirici, *J. Power Sources* **2023**, 557, 232534.

[25] J. Peng, H. Wang, X. Shi, H. J. Fan, *Adv. Mater.* **2024**, 37, 2410326.

[26] X. Shi, H. Wang, Z. Xie, Z. Mao, T. Zhang, J. Jin, B. He, R. Wang, Y. Gong, H. J. Fan, *Adv. Mater.* **2024**, 36, e2406794.

[27] H. Moon, A. Innocenti, H. Liu, H. Zhang, M. Weil, M. Zarrabeitia, S. Passerini, *ChemSusChem* **2023**, 16, e202201713.

[28] M. León, J. Silva, S. Carrasco, N. Barrientos, *Processes* **2020**, 8, 945.

[29] G. G. Stavropoulos, A. A. Zabaniotou, *Fuel Process. Technol.* **2009**, 90, 952.
